# Optimization of Invasion-Specific Effects of Betulin Derivatives on Prostate Cancer Cells through Lead Development

**DOI:** 10.1371/journal.pone.0126111

**Published:** 2015-05-12

**Authors:** Ville Härmä, Raisa Haavikko, Johannes Virtanen, Ilmari Ahonen, Hannu-Pekka Schukov, Sami Alakurtti, Enkhee Purev, Heiko Rischer, Jari Yli-Kauhaluoma, Vânia M. Moreira, Matthias Nees, Kirsi-Marja Oksman-Caldentey

**Affiliations:** 1 Industrial Biotechnology, VTT Technical Research Centre of Finland Ltd, Turku, Finland; 2 Division of Pharmaceutical Chemistry and Technology, Faculty of Pharmacy, University of Helsinki, Helsinki, Finland; 3 Turku Centre for Biotechnology BTK, University of Turku, Turku, Finland; 4 University of Turku, Faculty of Medicine, Institute of Biomedicine, Turku, Finland; 5 Process Chemistry and Environmental Engineering, VTT Technical Research Centre of Finland Ltd, Espoo, Finland; 6 National University of Mongolia, Ulanbataar, Mongolia; 7 Industrial Biotechnology, VTT Technical Research Centre of Finland Ltd, Espoo, Finland; Queensland University of Technology, AUSTRALIA

## Abstract

The anti-invasive and anti-proliferative effects of betulins and abietane derivatives was systematically tested using an organotypic model system of advanced, castration-resistant prostate cancers. A preliminary screen of the initial set of 93 compounds was performed in two-dimensional (2D) growth conditions using non-transformed prostate epithelial cells (EP156T), an androgen-sensitive prostate cancer cell line (LNCaP), and the castration-resistant, highly invasive cell line PC-3. The 25 most promising compounds were all betulin derivatives. These were selected for a focused secondary screen in three-dimensional (3D) growth conditions, with the goal to identify the most effective and specific anti-invasive compounds. Additional sensitivity and cytotoxicity tests were then performed using an extended cell line panel. The effects of these compounds on cell cycle progression, mitosis, proliferation and unspecific cytotoxicity, versus their ability to specifically interfere with cell motility and tumor cell invasion was addressed. To identify potential mechanisms of action and likely compound targets, multiplex profiling of compound effects on a panel of 43 human protein kinases was performed. These target de-convolution studies, combined with the phenotypic analyses of multicellular organoids in 3D models, revealed specific inhibition of AKT signaling linked to effects on the organization of the actin cytoskeleton as the most likely driver of altered cell morphology and motility.

## Introduction

Apart from skin cancer, prostate cancer (PrCa) is the most common cancer in men, especially in Europe. PrCa is typically hormone-dependent and the role of androgens in the progression from androgen-dependent to hormone-refractory PrCa is well established [1]. Early-stage PrCa can be successfully managed by operation alone and recurrent tumors are treated by ablation of circulating androgens by chemical castration. In contrast, late-stage castration-resistant PrCa (CRPC) with characteristic metastatic dissemination of the primary tumor, usually to the bone, remains the major cause of cancer-associated death. Although significant progress has been made in the treatment of primary and androgen-sensitive prostate tumors, curative therapies that specifically target the metastatic spread of advanced and aggressive CRPC currently do not exist. Partly, this is related to the lack of experimental model systems that faithfully represent the features of invasiveness and cell motility *in vitro* or *in vivo*.

Neither standard two-dimensional (2D) cell culture models (cell lines) nor animal models (mouse xenografts) accurately represent the full complexity of clinical tumors. 2D models particularly lack the tumor microenvironment (TME), the extracellular matrix (ECM), and stromal cell types. Such models only poorly correspond with drug sensitivity observed *in vivo* [2]. These functional shortcomings of pre-clinical drug discovery are reflected by the high attrition rates for cancer drugs (>95%) [3] in subsequent clinical studies. Thus, biologically more relevant *in vitro* tumor models need to be developed to improve productivity of drug discovery and reduce the number of animals required in drug development [4]. ECM preparations from different origins have proven particularly critical to investigate cell-cell interactions and differentiation in 3D culture [5]. Integrated, robust and standardized 3D platforms have become compatible for both high-throughput (HTS) and high-content screening (HCS) settings [2], and the efficient translation of 3D cell culture methods from basic research to industrial applications is ongoing [6]. Biologically relevant ECM such as collagens or laminin-rich basement membrane extracts like Matrigel are now widely used to study cellular mechanisms such as cell motility and invasion. The spectrum of multicellular morphologies formed by prostate epithelial and cancer cells in 3D cultures ranges from fully functional glandular acini, dysfunctional tumor spheroids; to invasive stellate structures [7,8] that lack most differentiated properties. Thus, the different morphology manifested in 3D lrECM culture is a solid indicator that correlates with various stages of malignant progression. Such biomimetic 3D cell models provide a powerful means to quantify cancer-related biological processes. Invasion and metastasis are the most critical hallmarks of cancer that can transform localized cancer into a life-threatening disease [9]. In 3D culture, tumor cell invasion is manifested either by single cells or cell aggregates that actively invade the surrounding ECM, using different modes of motility (e.g. amoeboid or collective invasion) [10]. PC-3 is one of the few PrCa cell lines that displays collective invasion characteristics both *in vitro* and *in vivo* [7,11]. Such complex multicellular processes cannot be reproduced in anchorage-independent, non-adherent 3D systems that lack biologically functional ECM, such as poly-HEMA [12], soft agar [13] or “hanging-drop” cultures of isolated spheroids [14]. A recent study showed that gene expression profiles of breast cancer cells cultured in 3D lrECM were much closer to *in vivo* than monolayer or poly-HEMA cultures [15]. High content screens (HCS) using lrECM-based platforms have been reported for prostate [16], breast [15], pancreatic [17], and ovarian cancers [18]. Such thoroughly standardized and miniaturized tissue-like models are required to systematically capture the effects of small molecule compounds/drugs, siRNAs, biological (e.g. antibodies and peptides), growth factors, or toxins on tumor biology. These complex biomimetic approaches are useful as faithful pre-clinical tools for investigating short- and long-term drug responses, therapy failure, or development of drug resistance [19]. Combined with high-content microscopic imaging and image-analysis methods, 3D phenotypic models can also be highly informative for lead discovery and lead optimization studies (LD or LO, respectively), in particular if the molecular drug target is unknown, and complex mechanisms such as tissue-specific differentiation, and cell-cell-interactions can be assessed based on unbiased, multiparametric read-out. Multiplexing of imaging-based readout also enables the simultaneous assessment of cytotoxicity, apoptosis, and effects on the cell cycle, e.g. by using appropriate fluorescent probes [2,20], thus reducing the need for excessive validation studies. In addition, real-time and live cell 3D assays based on high-content image analysis can be combined with endpoint studies addressing the expression of biomarkers, as described in previous publications [7,11].

Natural products (NPs) have been invaluable as tools for deciphering the logic of biosynthesis and as starting materials for developing front-line drugs [21]. Indeed, the majority of new chemical entities approved as drugs by the US Food and Drug Administration (FDA) have consistently been either NPs or NP-derived compounds [22]. The pentacyclic triterpenoids, secondary plant metabolites abundantly found in fruit peel, leaves and stem bark, have attracted great interest as therapeutic agents and dietary supplements [23,24]. In addition, semi-synthetic derivatives of the naturally occurring triterpenoids have been actively studied in search for new anticancer agents, with specific focus on anti-invasiveness properties [24–29]. Betulin and betulinic acid are lupane-type pentacyclic triterpenes abundant in the bark of birch species of the genus *Betula* L. [30]. Betulinic acid and other betulin derivatives have antiviral, anti-inflammatory, anti-malarial, and anti-cancer effects [31]. In addition, betulinic acid was identified as a selective inducer of apoptosis in melanoma cells [32], triggering a strong interest in triterpenes as anticancer agents. Moreover, betulin was found to block the invasive properties of brain and lung cancer cells, well below its cytotoxic concentration, suggesting a promising chemopreventive effect against metastases [33]. In the present study, we have used a combination of 2D and 3D PrCa cell models and HCS methods based on imaging and automated image analysis, to assess and validate the antineoplastic and anti-invasive properties of a library of 93 compounds. In this library, we have included the parental compounds betulin, betulinic acid (**2**) and their semi-synthetic derivatives, and compounds from another class of terpenoids with less studied biological effects, the abietanes. Overall, the library comprised 78 betulins and 15 abietanes.

## Results and Discussion

### Synthesis of a library of betulin and abietane derivatives

**Fig 1:**
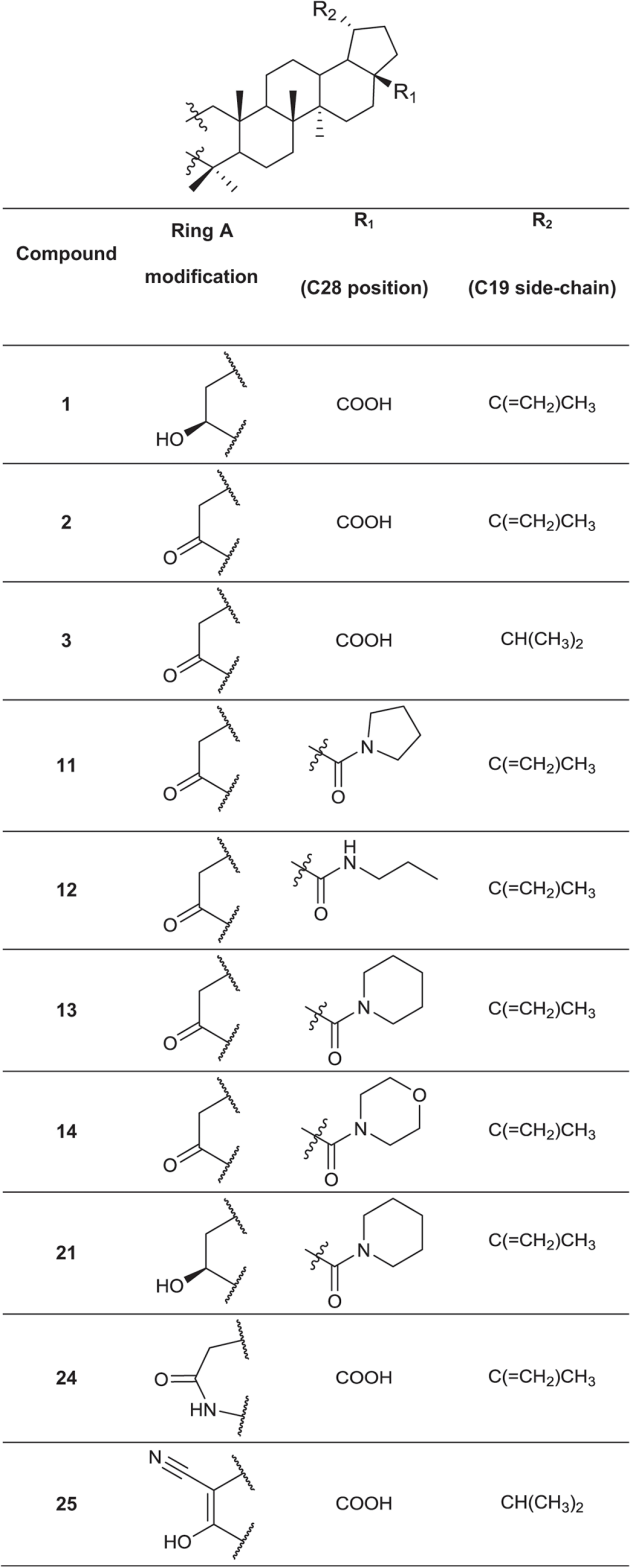
Chemical structures of the parental compound betulinic acid and its derivatives.

**Fig 2:**
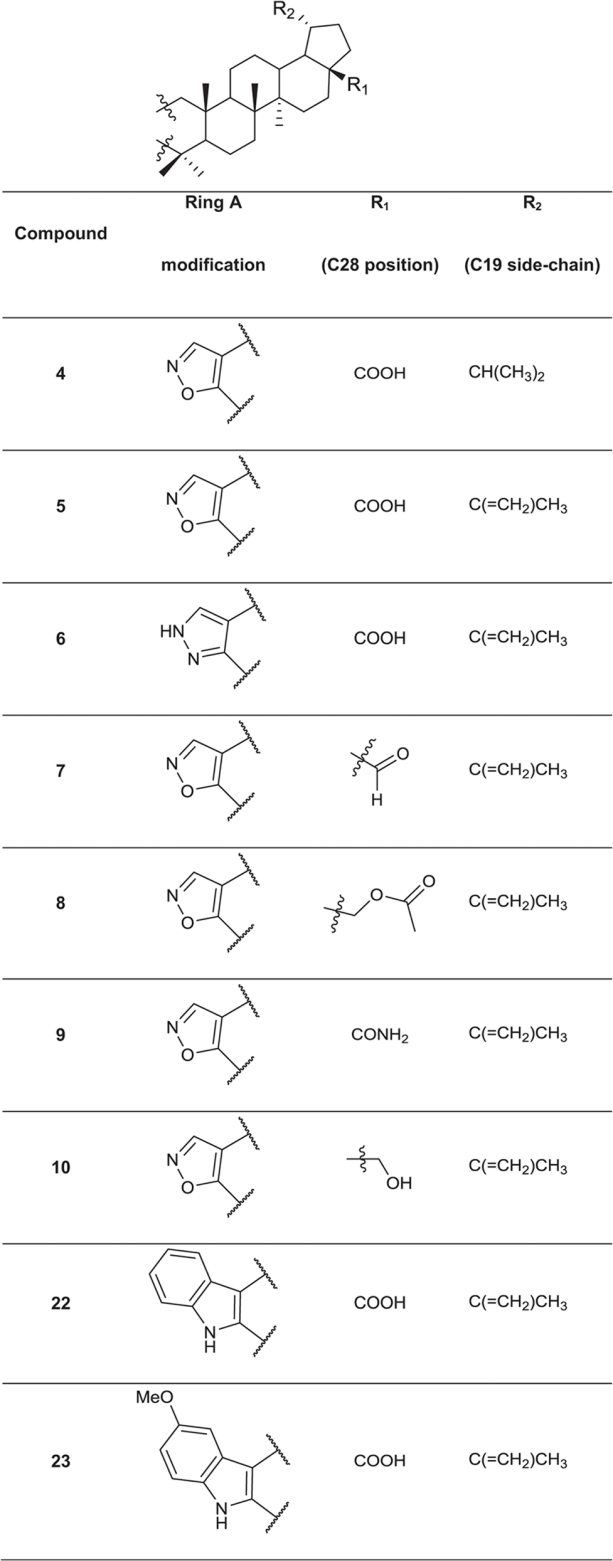
Chemical structures of the fused 5-membered ring derivatives of betulinic acid.

**Fig 3:**
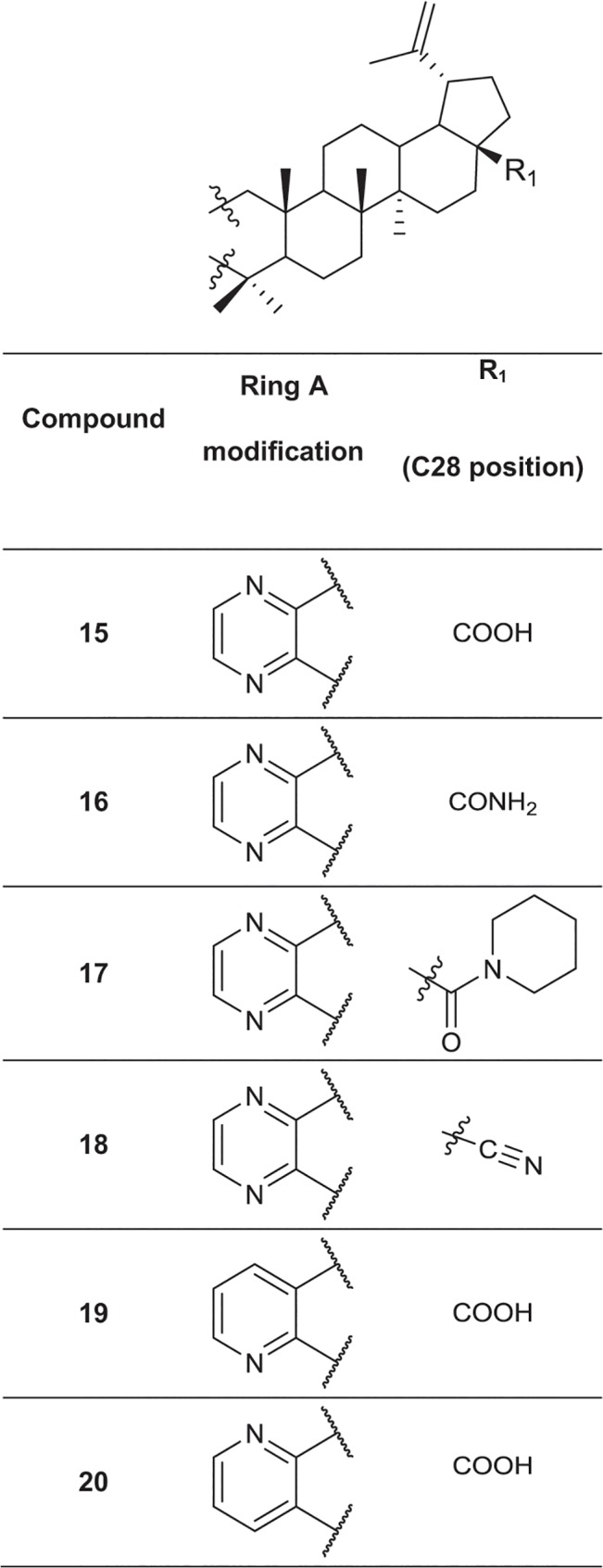
Chemical structures of the fused 6-membered ring derivatives of betulinic acid.

A set of 76 betulin derivatives was prepared starting from betulin and betulinic acid (**1**), whereas 13 derivatives were prepared from dehydroabietic acid and dehydroabietylamide (**Figs 1**–**3**, see also S1 File), using our expertise on natural products chemistry and following either our previously reported procedures or those of others [34–37]. Betulonic acid (**2**) was obtained from betulin by Jones oxidation, and used as a versatile intermediate for chemical syntheses. Different substituents were inserted into the positions 3 and 28 of the lupane core. Some derivatives were also prepared by fusing heterocycles to ring A, at positions 2 and 3. Other compounds contained one extra ring fused to rings C, D and E. Reduction of the isoproprenyl side chain of the triterpene core was done for some of the derivatives. Side-chain rearrangement resulted also in germanicane-type compounds. In the abietanes set the compounds prepared consisted mostly of urea and amide derivatives.

### High Throughput Screening (HTS) for bioactivity

These compounds were compiled as a library and biological activity was tested using 2D cell cultures in 384-well plates in high throughput screening (HTS) format (**S1 Fig**). The cell lines used were EP156T (non-transformed prostate epithelium), LNCaP (androgen sensitive PrCa), and PC3 (castration-resistant, invasive PrCa). The experiments were performed in three different compound concentrations (0.1, 1 and 10 μM) in triplicate, using CellTiterGlo as an end-point read-out for cell proliferation. A panel of 25 betulin derivatives was synthesized according to the most effective and cancer-specific compounds identified in the preliminary 2D screen, and selected for further studies.

### High Content Screening (HCS) for invasion-blocking activity

Next, these 25 most effective compounds were tested in 3D settings for specific anti-invasive properties. Three standard of care compounds widely used against PrCa were added as controls; namely, the androgen receptor antagonist enzalutamide (MDV3100), the C17α-hydroxylase/C_17,20_-lyase inhibitor abiraterone (Zytiga), and the classic mitotic inhibitor paclitaxel. Treatments started at day 4, when well-differentiated round acini were established. Compound exposure was then continued for six days, when most of the untreated multicellular structures had transformed into a highly invasive phenotype.

### Automated morphometric image analysis (AMIDA) and statistical evaluation

**Fig 4:**
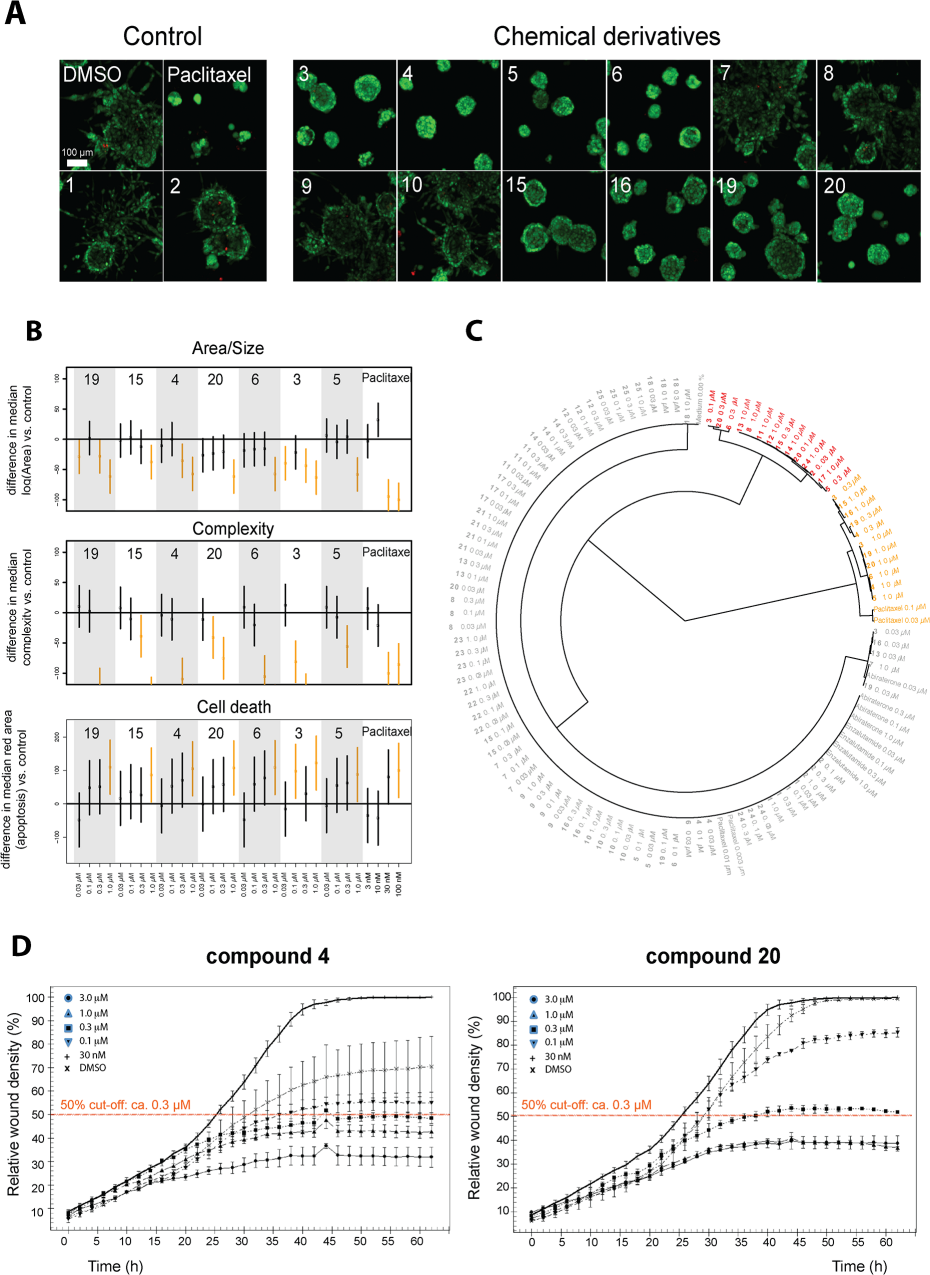
Primary 3D screen. PC-3 cells were cultured in 3D Matrigel ECM for 4 days and treated for 6 days with 25 betulin derivatives (from 2D high throughput screens), DMSO/vehicle control, and three reference compounds. A) Representative maximum intensity projections of confocal microscope stack images for selected compound treatments at 300 nM concentration (5× objective, scale 100 μm). B) Three graphs showing the relative impact on three morphometric parameters for 7 betulin derivatives, DMSO control, and one control compound (paclitaxel). Data scaling: displays the relative difference between median of Area/Complexity/Area Ratio, to DMSO control. Paclitaxel treatments and DMSO controls have been assigned values of -100 and 0, respectively. C) Hierarchical clustering was done using three morphological parameters derived from PC3 organoids: spheroid size (area), complexity and the number of dead cells. D) Wound healing curves of the two betulin derivatives **4** and **20**, highlighting the 50% cut-off level (orange dashed line).

The live 3D cell cultures were then stained with reactive dyes Calcein AM to detect living, and ethidium homodimer to detect dead cells. Images (**Fig 4A**) were acquired with confocal microscope and image data were analyzed with the AMIDA software [38]. The principles of morphometric image analysis are briefly outlined in **S2 Fig**.

**Fig 4A** shows representative confocal microscope images for some of the betulin derivatives. The highly effective, antimitotic control drug paclitaxel stood out even at very low concentrations (30 and 100 nM), showing a strong effect on all morphometric measures (**Fig 4B**): this resulted in smaller (reduced area), less invasive organoids (reduced complexity), with increased numbers of dead cells. The control compounds abiraterone and enzalutamide had no noticeable effect on PC-3 cells. Interestingly, **betulinic acid** (**1**) and **betulonic acid** (**2**) did not show significant anti-invasive effects, whereas compound **3**, bearing an isopropyl substituent at C19, was one of the most potent anti-invasive, growth inhibitory agents. Similarly, the isoxazole derivatives **4** and **5** displayed strong growth inhibiting and anti-invasive effects at lower concentrations (as low as 100 nM for **4** and 300 nM for **5**). All of these effective compounds differ only at position C19: compound **4** has an isopropyl side-chain, while compound **5** has the original isopropenyl side-chain. Furthermore, when the isoxazole ring of **5** was replaced with a pyrazole ring, the resulting compound **6** was even more potent and proved to be specifically anti-invasive. In contrast, the isoxazole derivatives with a formyl (**7**) or an acetoxy group (**8**), a primary amide (**9**) or a hydroxy (**10**) group at position C28 displayed negligible effects on the invasive phenotype. The relevance of the original carboxyl group at position C28 (betulonic acid, **2**) for anti-invasive or cytotoxic activity was studied by replacing this group with various amide groups. None of these compounds showed significant anti-invasive effects (**11**, **12**, **13**, and **14**) (not shown). Also compound **15** showed strong anti-invasive effects at 1.0 μM without any growth inhibition. However, when its carboxyl group at C17 was converted into a primary amide group, the resulting compound **16** had the same high level of anti-invasive effects as compound **15**. In contrast, derivatives with tertiary amide (**17**) or nitrile groups (**18**) at C17 were inactive (not shown). Two pyridine derivatives were also tested. No loss in anti-invasive activity was observed whether the pyridine nitrogen was adjacent to the C3 position of the original lupane skeleton (**19**), or next to the C2 position of the lupane skeleton (**20**). Interestingly, both compounds **19** and **20** were potently anti-invasive at significantly lower concentrations, compared to compound **15**.

Next, statistical analyses were performed to interpret the complex image data (**Fig 4B and 4C**). For simplicity, we focused on only three, basic morphologic parameters from the multiparametric AMIDA readout (Fig 4B): 1) **Area** (indicating growth of organoids), 2) structural **Complexity** (as a measure of the intensity of invasion), and 3) **Relative Area** of dead cells within organoids (red signal as a measure of cytoxicity or apoptosis). **Area** (Size of organoids; Fig 4B, upper panel) was measured as the number of pixels within a segmented structure. The logarithmic transformation of Area/Size data was used, which showed a close to Gaussian (normal) distribution within the images. Shape **Complexity** (Fig 4B, middle panel) was obtained using the structure size (area) and perimeter (number of border pixels). The more regular the shape of an organoid, the closer it is to a perfect circle, thus minimizing the perimeter relative to object size. We observed a close to linear dependency between log(Area) and log(Perimeter), indicating a natural (functional) relationship between these two features. Fitting a regression line to the data resulted in a useful estimate of the average perimeter of any organoid structure. Deviations from this average (residuals) were interpreted as a measure for the shape complexity of organoids. As a logical lower limit for this measure (represented by a perfect circle), we adjusted the intercept (height level) of the regression line to render all returned residuals as positive numbers. This simplified, rapid measure of the multicellular complexity indicated that the logarithm of residuals results in an almost symmetrical distribution of values. Hierarchical clustering, using these three basic measures from PC3 cultures yielded a simple dendrogram with two main branches: the first cluster included the most potent anti-proliferative and anti-invasive compounds (**Fig 4C**: yellow). The second cluster represents weak (Fig 4C: red) and non-effective (Fig 4C: grey) compounds.

### Validation of anti-invasive properties

Based on the primary screen, we selected six betulin derivatives for further validation of the anti-invasive effects by additional methods. In a standard wound healing assay performed in monolayer 2D culture, the efficacy of some compounds was strikingly different to 3D settings. For example, compounds **4,** and **20** completely reduced invasion in 3D already at 300 nM, respectively (**Fig 4A**), but showed only 50% reduction of wound closure after 64 hours (**Fig 4D**) at the same concentration. Also, compound **5** effectively inhibited invasive transformation of PC-3 spheroids already at 300 nM in 3D (**S3A Fig),** measured as “% roundness” retained after 10 days), but concentrations higher than 1 μM were required in 2D culture for comparable effects (**S3B Fig**). Also the most potent anti-invasive agents **6**, **16** and **19** (active at 100 nM in 3D conditions) reduced wound closure in 2D only by 50% at 3 μM and 1 μM, respectively (**S3B Fig**). Compound **21** is included here as an example for inactive derivatives. The fact that many compounds lacked the same potency in the conventional 2D monolayer culture suggests that the biological targets or pathways involved in cell motility are not equally active in cell migration on plastic surfaces. It is also likely that cell motility in 2D monolayer culture is controlled by other mechanisms than collective invasion in a 3D scaffold.

#### Secondary 3D screens

**Fig 5:**
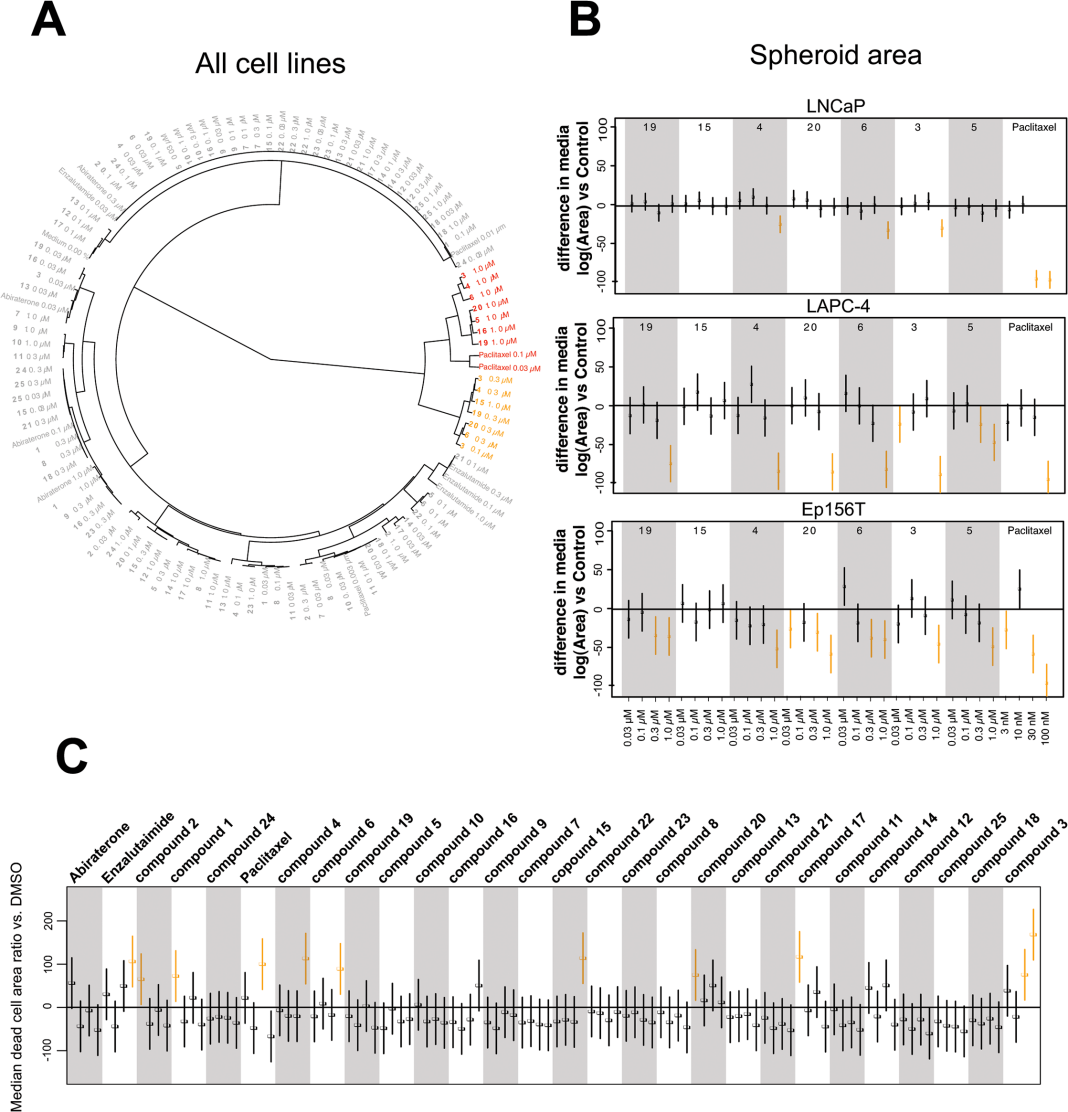
Secondary 3D screens. Experimental betulin derivatives and control compounds were tested across a panel of prostate cancer lines (LNCaP, LAPC-4) and non-transformed, prostate epithelial cells (EP156T) in 3D culture. **A**) The dendrogram is based on three main morphological parameters and combines all data from primary and secondary 3D screens. Effective compound treatments are indicated in red and yellow, yellow being the most invasion-specific. **B**) Boxplots showing the impact of selected compounds on spheroid size (Area). Data scaling: as described in Fig 4 **C**) Effects of all betulin derivatives and control compounds on cell death (number of dead cells).

Next, the 25 selected betulin derivatives depicted in **Figs 1–3** including control compounds were again thoroughly tested across the same panel of prostate-derived cell lines already used in the preliminary, 2D high-throughput screen. The resulting dendrograms were either generated separately for each cell line (**S4 Fig**), combined into a single dendrogram (**Fig 5A**), or shown as reduced organoid sizes (area; **Fig 5B**). In the non-invasive, but hormone-sensitive LNCaP organoids, most compound effects were relatively mild. Non-effective treatments comprise the vast majority of the graph (**S4 Fig**: grey), while the few effective compounds fall into two connected branches (yellow and red). As expected, the androgen antagonist enzalutamide but not abiraterone were among the effective treatments. Paclitaxel clusters together with betulin derivatives at higher concentrations—these compounds were also effective on hormone resistant, invasive PC-3 cells. The dendrogram for hormone dependent, non-invasive LAPC-4 (**S4B Fig**) again demonstrates the efficacy of all positive controls (abiraterone, enzalutamide and paclitaxel; within yellow and red clusters). The most active betulin derivatives again fall inside these same clusters (3, 4, 6, 16, 19 and 20) adjacent to positive controls. In contrast to PrCa cell lines, the non-transformed, normal-like Ep156T cell line (**S4C Fig**) also responded to betulonic acid (**2**), at least at the highest concentration. In general, as with LNCaP and LAPC4 cells, the effects on Ep156T growth were mild (**Fig 5B**).

In order to assist functional categorization of our betulin derivatives, we combined all data into a single dendrogram, shown in **Fig 5A**. In addition, multiparametric representation of all experimental data as a single heatmap is highly informative (**S5 Fig**) and a potent way to illustrate the effects of compounds on growth, structural complexity and cell death across multiple cell lines. We divided the compounds and controls into three clusters: 1) growth inhibitory compounds, 2) strong and weak anti-invasive compounds, and 3) inactive compounds. The morphological effects in 3D culture are summarized in **S6 Fig** The most potent anti-invasive effects (on PC-3 cells) were shown by compounds **3**, **4**, **6**, **15**, **19** and **20**, at concentrations of 300 nM or lower (with the exception of compound **15**), with no noticeable effects on the other cell lines. Most of these compounds, however, showed growth-inhibitory effects in all cell lines at concentrations of 1 μM or higher. In conclusion, many betulin derivatives are cytotoxic and inhibit cell growth at high concentrations (>1 μM). However, at lower concentrations, these compounds may act as potent and specific inhibitors of cell invasion and motility.

#### Estimation of anti-invasive vs cytotoxic activity

**Fig 6:**
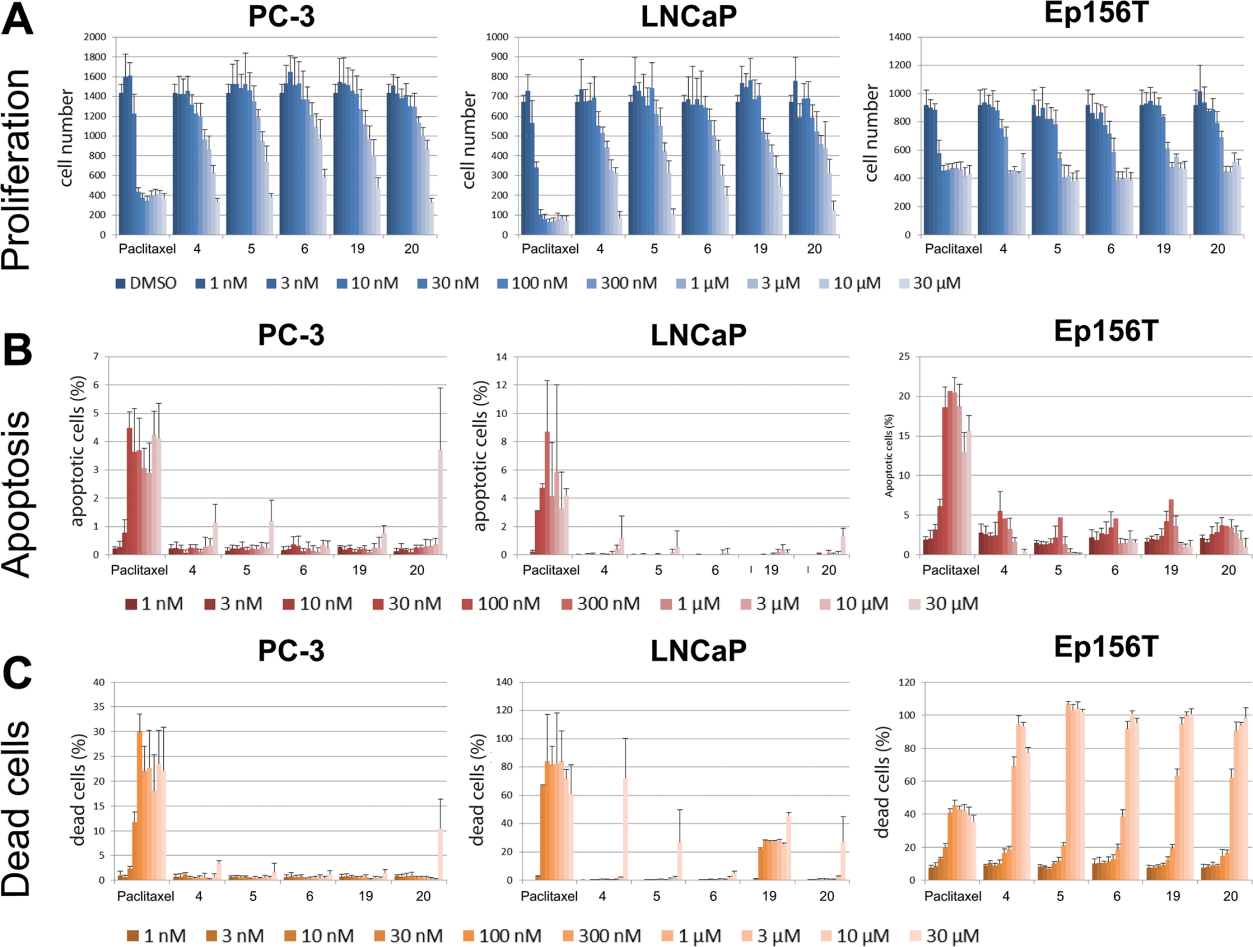
Cytotoxicity tests performed in 2D monolayer culture. **A**) Cell proliferation/cell number, **B**) programmed cell death (apoptosis), and **C**) number of dead cells were assessed by conventional assays, combined with high-content microscopy (Operetta). Cells were treated with the five most effective betulin derivatives, including paclitaxel control for 72h. Proliferation was measured as the total number of nuclei (= cells), apoptosis as ratio of caspase-3 positive versus all cells, and cell death as ethidium homodimer-2 positive nuclei versus all cells.

To rule out that cytotoxic and antimitotic effects were misinterpreted as anti-invasive, we tested compounds 4, 5, 6, 19 and 20 for inhibition of proliferation and induction of cell death in a 2D monolayer culture (summarized in **Fig 6**). A broad range of compound concentrations (1 nM to 30 μM) were used for PC-3, LNCaP and Ep156T cell lines, and analyzed with the PerkinElmer Operetta high-content imager. In PC3 cells, most compounds did not show noticeable effects on proliferation at concentrations lower than 10 μM (**Fig 6A**). LNCaP cells were more sensitive, with growth inhibition/cytotoxicity starting at 3 μM. The effects on non-transformed, apoptosis-sensitive Ep156T cells were strongest. Here, cytotoxic effects were typically observed already at 0.3–1 μM. None of the compounds specifically induced cell death in PC-3 or LNCaP cells at concentrations below 30 μM (**Fig 6B and 6C**; except for compound 19). We further calculated EC_50_ values for proliferation, apoptosis and cell death for each compound, using the PerkinElmer Harmony software (method validation shown in **S7 Fig**). The only assay that produced results consistent with those of the betulin derivatives tested in the 2D cell culture model was the proliferation assay (**Table 1**).

**Table 1:** Estimated EC_50_ values for cell proliferation.

	PC-3	LNCaP	EP156T
Compound **6**	90.8	6.4	0.21
Compound **5**	9.5	64.6	0.22
Compound **4**	55.9	93.1	0.2
Compound **19**	86.9	0.9	0.18
Compound **20**	66.9	131.5	0.19
**Paclitaxel**	0.0012	0.0006	0.0008

Values have been calculated using Harmony High-Content Imaging and Analysis Software.

### Effects on cell cycle progression and mitosis

We next tested if the betulin derivatives had specific effects on cell cycle progression and mitosis at concentrations of 300 nM (not shown) and 1 μM in 2D culture (**Fig 6D** and **S8 Fig**). There were no noticeable differences in cell cycle progression in response to any of the tested compounds. We also assessed cell proliferation and mitosis by measuring PCNA and cyclin B1 protein levels (**S8B Fig**). As expected, betulin derivatives showed no effect on the expression of either protein at the concentration tested. These findings again support that betulin derivatives are only cytotoxic or cause DNA damage at high concentrations (PrCa EC_50_ values ranging from 1–90 μM), whereas they promote considerable anti-invasiveness effects at low nanomolar concentrations.

#### Possible mechanism(s) of action of the betulin derivatives

**Fig 7:**
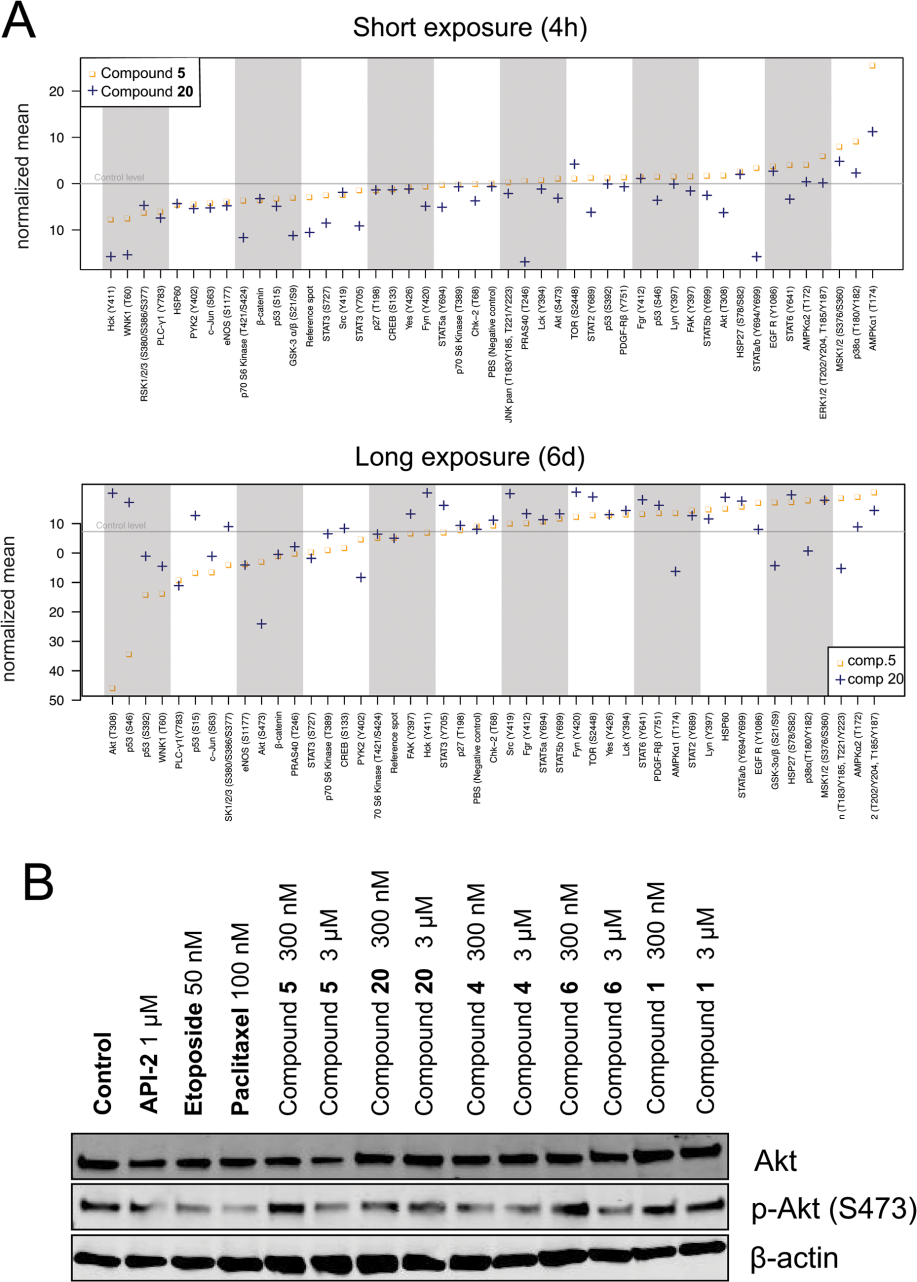
Mechanisms of action. PC-3 spheroids were exposed to two representative betulin derivatives, **5** and **20**, for 4 h (at 1 μM) and 6 days (at 0.3 μM). A) Computational quantification of signal intensities from the kinase arrays, aligned by fold changes observed (left to right). B) Western blot of total and phospho-Akt (S473) shown for both short and long-term exposure to **20** and **5**.

In order to elucidate possible mechanism of action for our betulin derivatives, we used a phospho-kinase array that quantitatively detects the phosphorylation levels of 43 kinases in cell lysates (**Fig 7**). Lysates were extracted from 3D cell cultures of PC-3 cells, and exposed to betulin-derived compounds for short (4 hours) or long-term (6 days) periods. For these studies, we selected the two representative derivatives **5** and **20** as the most specific, least cytotoxic inhibitors of cell invasion. The short-term (4 h) exposure was performed at a relatively high concentration of 1 μM, whereas for the long-term (6 d) exposure, a concentration of only 300 nM was used; both well below the cytotoxic EC_50_ values (10 μM for compound **5** and 67 μM for compound **20**). The kinase arrays were also quantified by image densitometry (**Fig 7A**). Phosphorylation levels of four kinases, in both short and long-term exposures, were clearly decreased: STAT3 (Y727), c-Jun (S63), eNOS (S1177) and PLC-γ1 (Y783) (**Fig 7A** top and bottom panels). Both compounds also reduced p53 (S15) phosphorylation in the short-term exposure and p70 S6 kinase (T389), p53 (S392) and PYK2 (Y402) phosphorylation in the 6-d exposure, and increased AMPKα1 (T174) phosphorylation. Compound-specific effects for substance **20** included differential phosphorylation of Hck (Y411), pan-JNK, GSK-3α/β (S21/S9), STAT3 (Y705) and WNK1 (T60) sites. Akt (S473) phosphorylation was only inhibited by the 6-d exposure. Compound **20** also increased the phosphorylation of mTOR (S2448), Fyn (Y420) and Src (Y419). In contrast, compound **5** did not cause any specific changes in kinase activity in the short-term exposure, and showed only few distinct changes in the long-term treatment. Most notably, Akt (T308) phosphorylation was also abolished almost entirely in response to exposure with compound **5**, whereas the phosphorylation of p53 sites S46 and S392 were consistently decreased (**Fig 7A**). The reduction of AKT activity by both compounds was validated by western blotting, using an independent, p-AKT specific antibody (**Fig 7B**). The short treatments caused only modest changes with a maximum reduction of protein phosphorylation by 2-fold (20: GSK-3α/β S21/S9 and STAT3 Y705). Phosphorylation levels of only three kinases were altered more than 2-fold in the long-term exposures, namely AKT (T308), p53 (S46) and eNOS (S1177), caused by nanomolar concentrations of compound **5**. Compound **20** had much less dramatic effects on kinase phosphorylation when compared to compound **5**, despite its generally more pronounced anti-invasive effects. In summary, the observed reduced AKT and p53 activity as well as the altered phosphorylation levels of several proteins including eNOS, pan-JNK, and GSK-3α/β, suggest decelerated cell metabolism or decreased cell viability.

**Fig 8:**
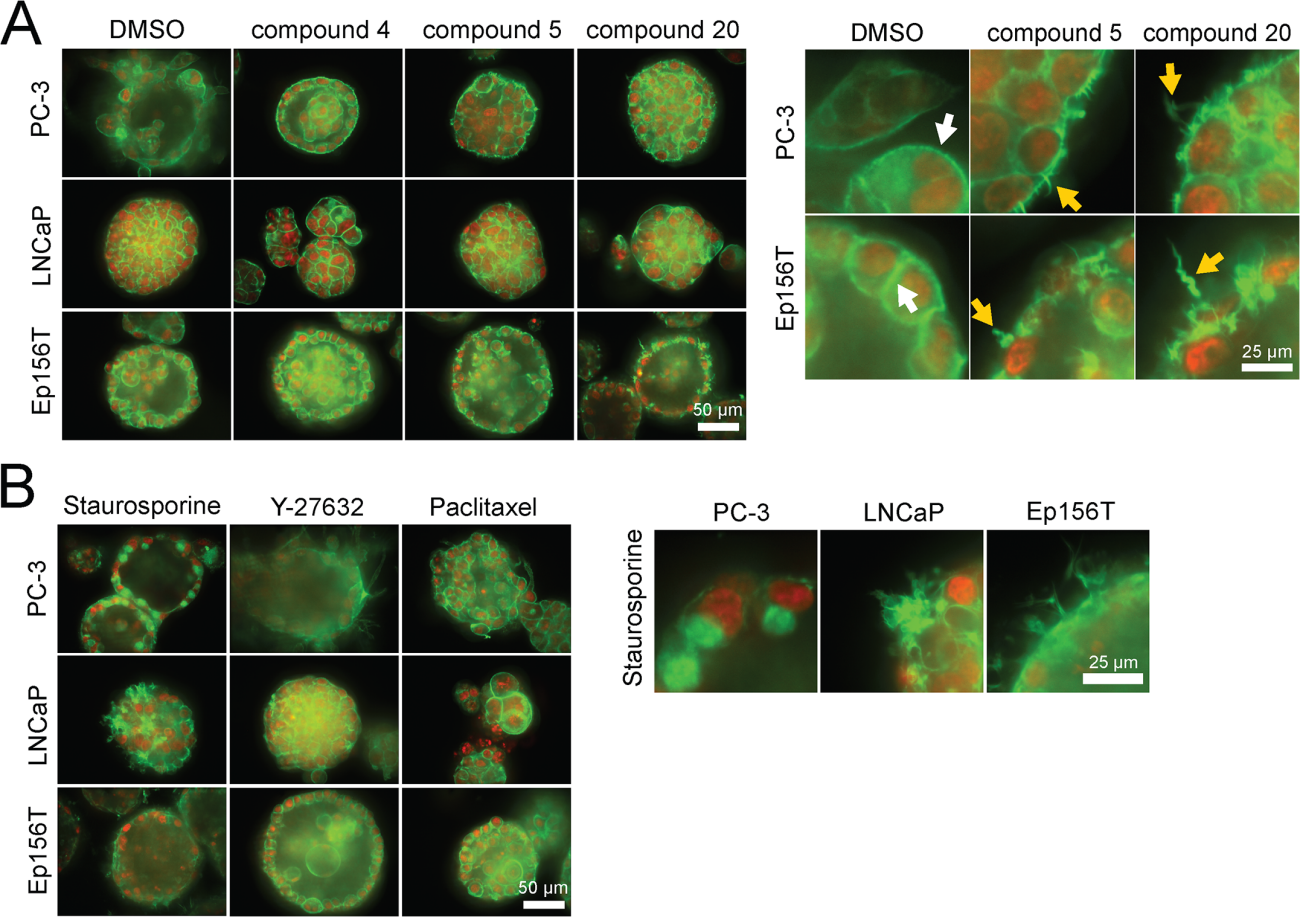
3D morphologies of prostate spheroids, exposed to selected betulin derivatives and captured with a confocal microscope. Actin cytoskeleton (filamentous or F-actin) is stained green (phalloidin), nuclei with a red dye (confocal microscope images, 40× objective, scale bar shown for each panel on the right lower corner).

We also explored the 3D multicellular morphologies of PC-3, LNCaP and Ep156T spheroids exposed to the 5 selected betulin derivatives both for short and long periods of 4 hours and 6 days. From a detailed analysis of the F-actin cytoskeleton (phalloidin staining), it became evident that compounds **5, 6, 15** and **20** effectively disrupted the organization of actin cytoskeleton after 48 hours of exposure (**Fig 8A**). Interestingly, an almost identical actin “corkscrew” phenotype was seen in the normal Ep156T acini and PC-3 spheroids (**Fig 8A**: magnification panel on the right) whereas the typical cortical actin organization in LNCaP spheroids remained intact. This suggests that these betulin derivatives may target relevant pathways in acinar morphogenesis and dynamic processes involved in cell motility (also EP156T cells form dynamic “branching” structures that penetrate the ECM). For phenotypic comparisons, we tested a panel of reference compounds targeting Akt, Rac, ROCK, Src, and other pathways, broadly linked to the actin cytoskeleton. None of the tested control compounds affected the cytoskeleton in a comparable fashion, with the exception of the non-specific, pan-kinase inhibitor staurosporine. However, these effects were not uniform across the panel of cell lines (**Fig 8B**, magnification on right).

As a control, we also tested morphologies treated with the same panel of anti-invasive compounds **4, 5, 6, 16** and **20** as well as betulin (**1**) on PC3 cells grown in 2D monolayer cultures (**S9 Fig**). No morphologies and visible effects on the actin cytoskeleton as seen in 3D settings were observed in 2D growth conditions. In contrast, blocking Rac (IPA3 compound) or Rho Kinase/ROCK signaling (Y-27632) did result in a clear rounding (IPA3) or elongation (Y-27632) of single PC3 cells. This indicates that mechanisms affecting the actin cytoskeleton and cell motility by particularly anti-invasive betulin derivatives may a) not apply in 2D conditions and b) may not function via the Rho and Rac signaling pathways.

## Conclusions

The morphological and functional effects of a diverse set of betulin and abietane derivatives on a selected panel of prostate cancer cell lines were analyzed using both routine 2D and, for a focused panel of 25 betulin derivatives, organotypic 3D cell culture models, by image-based high-content analysis. Our data highlighted the dose-dependent, potent and robust anti-invasive activity of some betulin derivatives at nanomolar concentrations, with minimal cytotoxicity. Compounds bearing heterocyclic rings fused to ring A including pyrazine, pyrazole, oxazole, indole, and pyridine moieties, were among the most promising in suppressing PC-3 cell invasiveness. A free carboxyl group at C28 was important for their activity, which was considerably improved when compared to the parent betulinic acid. Kinase phosphorylation profiling, performed for two representative betulin derivatives (**5** and **20**), suggested that these compounds do not primarily affect cell cycle progression and mitosis, but induce cytotoxic stress only at higher concentrations and after long exposure times, as indicated by p53 de-phosphorylation. Direct evidence for DNA damage was not found. Both compounds reproducibly decreased AKT phosphorylation. In line with the effects on AKT phosphorylation, we noticed that many betulin derivatives, including **5** and **20**, effectively disrupted actin cytoskeleton organization, resulting in a peculiar corkscrew-like phenotype of the filamentous actin. This mechanism may be causally linked to the efficient suppression of the invasive properties of PC-3 cells in both 2D and 3D conditions. Overall, our findings suggest that betulin-derivatives such as **5** and **20** may specifically target cell motility and invasion by affecting the organization of filamentous actin fiber network at low nanomolar concentrations, without significant cytotoxic effects. Our study greatly contributed towards establishing the true biological effects of betulin derivatives on prostate cancer cells, with focus on invasiveness, by integrating chemical synthesis with 3D screening platforms. They also highlight the role of betulin and betulinic acid as leads for the development of potent and specific anti-invasive agents. The implementation of these platforms in drug discovery programs could significantly contribute towards finding more selective and thus less toxic treatments for cancers where metastasis is particularly relevant such as those of the prostate.

## Materials and Methods

### Compound synthesis

Chemical synthesis and characterization data of the betulin and abietane derivatives is described elsewhere, except for three novel compounds. The chemical synthesis and characterization of the novel compounds and chemical structures of all the other compounds, which are not included in **Figs 1–3**, are described in detail in S1 File. Chemical formulas of the most potent 25 betulin derivatives are shown in **Figs 1–3**, whereas the other betulin and abietane derivatives screened in this study are depicted in the Supporting Information (including additional figures).

### Cell lines and culture conditions

Cell lines were obtained from American Type Culture Collection (PC-3 and LNCaP, Manassas, VA, USA) or originator laboratories (EP156T; Varda Rotter, Rehovot, Israel). PC-3 and LNCaP cell lines were propagated in RPMI-1640 medium (Sigma-Aldrich, St. Louis, MO, USA), EP156T cells were cultured in Keratinocyte Serum-Free Medium (KSFM; Invitrogen, Carlsbad, CA, USA), supplemented with 50 mg/L bovine pituitary extract, 5 mg/L epidermal growth factor (EGF) and 2% fetal bovine serum (FBS) for 3D conditions.

#### Primary cell-based screens in 2D culture

Cell culture in 384-well plate format and high-content screening were performed as described in [16].

#### Cell-based screens in 3D culture

All 3D cultures were done in growth factor-free Matrigel Basement-membrane Matrix (Corning Inc., New York, NY, USA) using 96-well Angiogenesis μ-plates (ibidi GmbH, Munich, Germany) as described before.[38] “Sandwich” assays in short: bottom wells of cooled Angiogenesis plates were filled with 10 μL 4 mg/mL Matrigel, centrifuged for 20 min 200 *g* and incubated at 37°C temperature for approximately 30–60 min or until the ECM had polymerized. 1,000–1,500 cells were mixed in 2 mg/mL ECM-medium, and 20 μL of cell suspension was added in each well. The plates were centrifuged for 10 min at 100 *g*, or until the cells had settled on top of the lower ECM. Finally, the outer wells and side reservoirs were filled with water for humidification, and the culture plates placed into the incubator for 3–4 h or overnight. The following day, 60 μL of cell culture medium were added; refreshed every third day by carefully aspirating the medium. This “sandwich” setting allows almost unifocal alignment of cells and 3D structures in a single optical plane, thus reducing the time required for confocal stack imaging.

### Morphometric image analysis (AMIDA), data normalization, and mathematical/statistical modeling

Automated image analyses were essentially performed as described previously (S9 Fig).[38] Statistical data processing and mathematical modeling of treatment responses are described in depth in S1 File.

### Chemicals and compound treatments

All control compounds were purchased from Selleck (Munich, Germany), except for staurosporine (Sigma-Aldrich, St. Louis, MO, USA) and Y-27632 (Tocris, Bristol, UK) and dissolved in dimethyl sulfoxide (DMSO) as a vehicle at 10 mM. In the primary and secondary 3D screens, experimental and control compound exposures were performed in triplicates. Four concentrations for each compound were applied (0.03, 0.1, 0.3 and 1 μM). Compound treatments were initiated four days after cell embedding, and continued for six days after which spheroids were stained and imaged.

### Image acquisition and pre-processing

3D cell cultures were double-stained with calcein AM fluorescent dye (Molecular Probes, Eugene, OR, USA) and ethidium homodimer-2 (Invitrogen, Carlsbad, CA, USA). Confocal images were acquired with a Zeiss Axiovert-200M microscope, equipped with Yokogawa CSU22 spinning disc confocal unit using Zeiss Plan-Neofluar 5× objective. Intensity projections were created with SlideBook (Intelligent Imaging Innovations Inc., Denver, CO, USA). Background noise was removed by normalization, using either SlideBook or ImageJ (NIH, Bethesda, MD, USA) programs.

### Wound healing assay

Cells were cultured on ImageLock plates (Essen Bioscience, Ann Arbor, MI, USA) until fully confluent and scratched with a WoundMaker instrument (Essen Bioscience). All detached cells were removed by aspiration and medium supplemented with experimental compounds was added. Wound closure was monitored and quantified with the IncuCyte live-cell imager (Essen Bioscience).

### Proliferation, apoptosis and cell death assays

Cells were transferred into CellCarrier 384-well plates (PerkinElmer, Waltham, MA, USA) at a density of 1250 cells/well, using Multidrop dispenser (ThermoFisher Scientific, Waltham, MA, USA). After overnight incubation at 37°C experimental, compounds were added with an ATS Acoustic Transfer System (EDC Biosystems, Fremont, CA, USA). For Ep156T cells the protocol was done in reverse with compounds dispensed before seeding of cells. Fluorescent markers were dispensed in culture medium using ATS system after 72-h compound exposure. Nuclei were stained with cell-permeable Hoechst 33342 (Molecular Probes, Eugene, OR, USA), dead cells with ethidium homodimer-2 (Invitrogen, Carlsbad, CA, USA) and apoptotic cells with NucView caspase-3 detection reagent (Essen Bioscience, Ann Arbor, MI, USA). Cells were imaged with Operetta high-content imager (PerkinElmer, Waltham, MA, USA). Proliferation was measured from the number of nuclei (cells), cell death and apoptosis from positive cells/total cells ratio using Harmony image analysis software (PerkinElmer, Waltham, MA, USA). EC_50_ values were also assessed with the Harmony software.

### Cell cycle and DNA damage response analyses

Propidium iodide–based cell cycle analysis method was adapted from Moores Cancer Center protocol (UC San Diego). In short: cells treated for 72 h with experimental compounds on 6-well plates were washed, detached with trypsin, transferred into Eppendorf tubes, washed first with medium and ice cold phosphate buffered saline, pH 7.4 (PBS). Between each step, cells were spun down (200 *g* × 5 min at 4°C). Finally, cells were suspended in 0.5 mL ice cold PBS and slowly added to 1 mL ice cold absolute ethanol. Pellets were stored at -20°C for at least 72 h, after which they were washed with PBS and stained with 50 μg/mL propidium iodide (Molecular Probes, Eugene, OR, USA). DNA content of 10,000 cells/sample was measured using a fluorescence-activated cell sorter BD FACSCalibur (BD Biosciences, Franklin Lakes, NJ, USA). Cell cycle analyses were performed using the FCS Express 4 software (De Novo Software, Los Angeles, CA, USA).

### Antibody arrays

In order to generate sufficient protein material for antibody arrays, we resorted to 6-well Millicell hanging cell culture inserts (Merck Millipore, Billerica, MA, USA). Membranes were pre-coated with 4 mg/mL GFR Matrigel (Corning Inc., New York, NY, USA) and incubated at 37°C for 1 h to prevent attachment to the membrane. GFR Matrigel (2 mg/mL) was prepared and 100,000 cells were added in. The Matrigel-cell suspension was transferred into the coated wells and incubated overnight at 37°C. Fresh medium was added the following day under the insert. After four days in culture fresh media with compounds **20** and **5** at 0.3 μM concentration compounds were added for six days long-term exposure. On day 10, the same compounds were added in fresh medium at 1 μM concentration for the 4h short-term exposure. DMSO was used as a negative control in both exposures. Cells were harvested by first washing the inserts with ice cold PBS, then incubating the isolated ECM slabs in ice cold 5 mM ethylenediaminetetraacetic acid (EDTA) in PBS on ice on a table top rocker for 45 min to dissolve the ECM, and finally by washing the cell pellets once with PBS before lysing them in Lysis Buffer 6 (R&D Systems, Minneapolis, MN, USA). The antibody arrays used were from the Human Phospho-Kinase Array Kit (R&D Systems, Minneapolis, MN, USA). The samples were assayed according to manufacturer’s instructions. Exposed radiograms were scanned with a tabletop scanner and spot intensities quantified using Array-Pro Analyzer (v4.5.1.73) software.

## Supporting Information

S1 Fig Summary of the results from High Throughput Screening of betulin derivatives and abietanes in 2D cell culture conditions (384-well format). Screening data for cell lines PC3, LNCaP and EP156T were measured as CellTitreGlo intensity, and normalized values were combined into a single plot. Compounds named according to internal nomenclature. The running number for compounds selected for further experiments is shown in parentheses.
(EPS)

S2 Fig Morphometric image analysis. **A**) Maximum intensity projections of two confocal image stacks segmented with AMIDA image analysis program. The left image shows PC-3 cells in their invasive phase cultured 10 days in 3D Matrigel ECM. The right image shows chemically suppressed invasion. **B**) A random sample of thousand observations plotted from the complete data set and a robustly fitted regression line close to linear dependency indicating the natural relationship between two morphological features log(Area) and log(Perimeter). Deviations from this average (residuals) can then be interpreted as a measure for the shape complexity. **C**) A random sample of 40 structures ordered based on the complexity measure.
(TIF)

S3 Fig Validation of anti-invasion effects of betulin derivatives. **A**) Graph showing the transition of PC-3 spheroids from symmetrical acini to irregular invasive structures over 6 days, as measured by general symmetry (roundness %). Betulin derivatives **5** (left) and **20** (right) suppress the invasive transformation at concentrations over 300 nM. **B**) Conventional wound healing assay performed in monolayer culture over a period of 64 hours. Wound closure is measured as relative wound density i.e. percentage of original wound area reclaimed by migrating cells. 50% inhibition serving as a cut-off point for effectiveness is highlighted with an orange dashed line.
(EPS)

S4 Fig Secondary screens. Dendrograms for **A**) LNCaP, **B**) LAPC-4, and **C**) Ep156T betulin screens in 3D culture have been constructed using three main read-outs: spheroid size, spheroid complexity, and cell death. Clusters highlighted with yellow and red color represent the most biologically active compounds.
(EPS)

S5 Fig Primary and secondary screens. The heatmap shows morphometric data from betulin screens performed with four cell lines (PC-3, LNCaP, LAPC-4, Ep156T). The main read-outs (Area = Size, Complexity = Invasiveness, Red = Cell death) are shown in their own columns. Data scaling: DMSO control has been given a value 0 whereas paclitaxel control is valued -100 or 100 depending on the read-out. Color key is located in the upper right corner.
(EPS)

S6 Fig Confocal images from primary and secondary betulin screens. Viable cells have been stained with calcein AM (green) and dead cells with ethidium homodimer-2 (red) (5× objective, maximum intensity projections, scale bar = 100 μm).
(TIF)

S7 Fig Positive control paclitaxel EC_50_ curves for proliferation, cell death and apoptosis in monolayer culture. EC_50_values, calculated with PerkinElmer Harmony software, are displayed in each graph.
(TIF)

S8 Fig Cell cycle, proliferation and mitotic analyses. **A**) Histograms for DNA content for the cell lines PC-3, LNCaP and Ep156T; exposed to three betulin derivatives **4**, **5** and **20** at 1 μM concentration for 72 h in monolayer culture. Relative proportions of each cell cycle phase (G1, S and G2), assessed using Flowing software (v2.5.1), are displayed next to each histogram (in %). **B**) Expression of proliferating cell nuclear antigen (PCNA) and mitotic cyclin B1 protein in response to 72h exposure to betulin derivatives. 24h paclitaxel treatment was used as mitotic arrest control.
(TIF)

S9 Fig Morphologies of PC-3, LNCaP and Ep156T cells in 2D monolayer culture, exposed 72h to selected betulin derivatives. Actin cytoskeleton (filamentous or F- actin) is stained green (LifeAct), nuclei with a red dye (confocal microscope images, 40× objective, scale bar shown for each panel on the right lower corner).
(TIF)

S1 File Supplemental Methods. (DOCX)
