# Supplementary figures and images for: Optimization of Invasion-Specific Effects of Betulin Derivatives on Prostate Cancer Cells through Lead Development
Source: PLoS One. 2015 May 12;10(5):e0126111. doi: 10.1371/journal.pone.0126111 (PMC4428838; doi:10.1371/journal.pone.0126111)

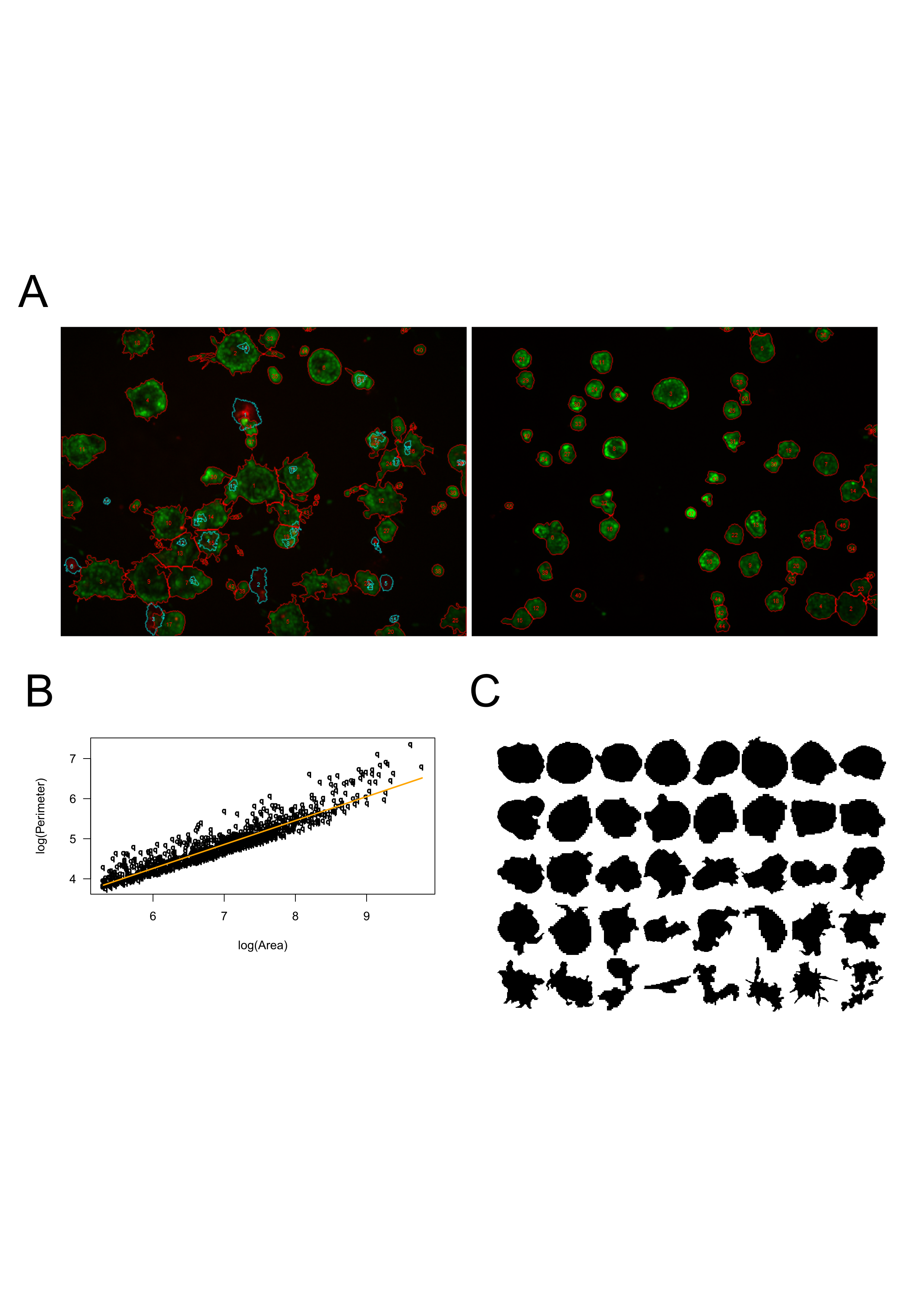

Supplement: S2 Fig — A) Maximum intensity projections of two confocal image stacks segmented with AMIDA image analysis program. The left image shows PC-3 cells in their invasive phase cultured 10 days in 3D Matrigel ECM. The right image shows chemically suppressed invasion. B) A random sample of thousand observations plotted from the complete data set and a robustly fitted regression line close to linear dependency indicating the natural relationship between two morphological features log(Area) and log(Perimeter). Deviations from this average (residuals) can then be interpreted as a measure for the shape complexity. C) A random sample of 40 structures ordered based on the complexity measure. (TIF) [file pone.0126111.s002.tif]

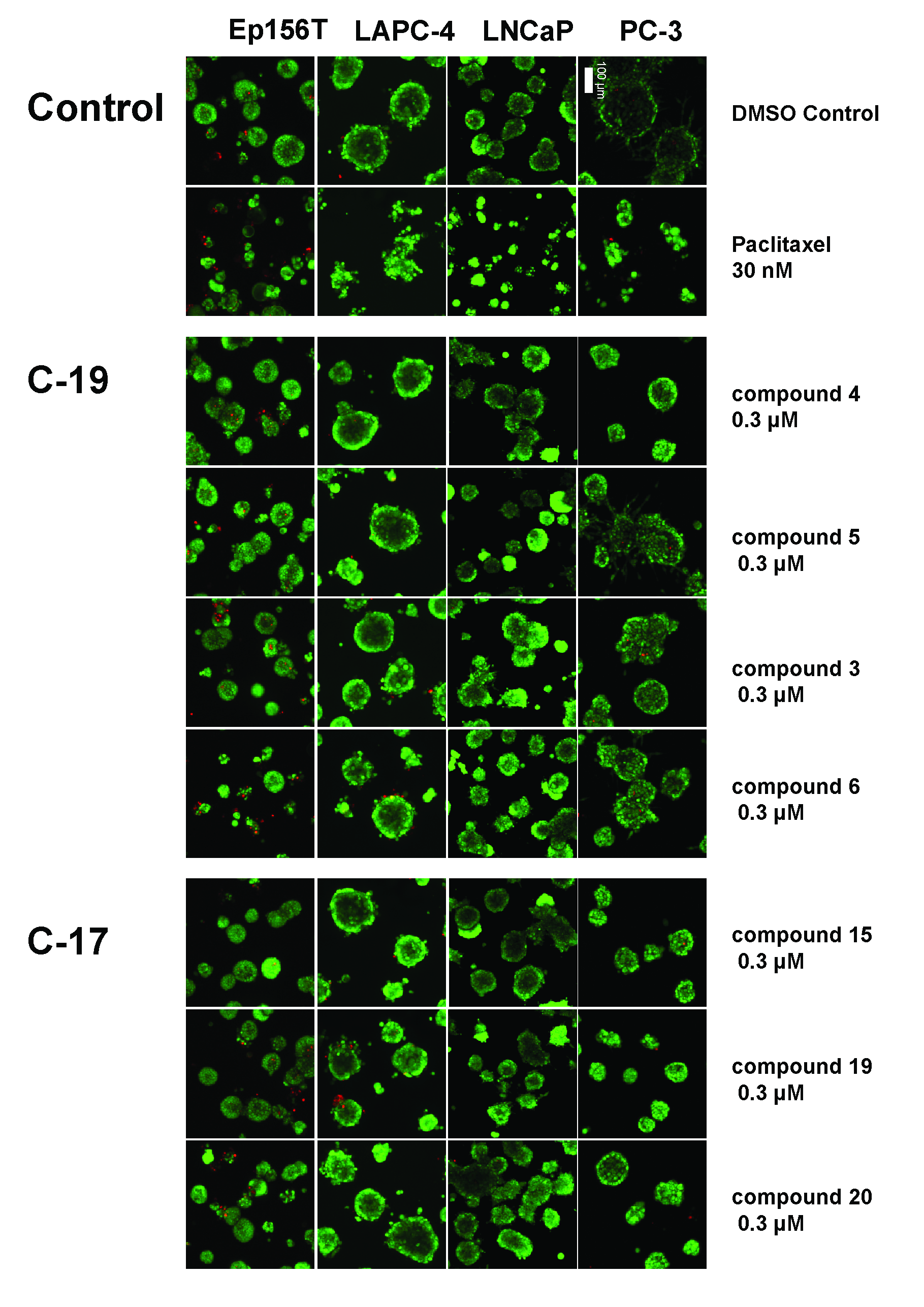

Supplement: S6 Fig — Viable cells have been stained with calcein AM (green) and dead cells with ethidium homodimer-2 (red) (5× objective, maximum intensity projections, scale bar = 100 μm). (TIF) [file pone.0126111.s006.tif]

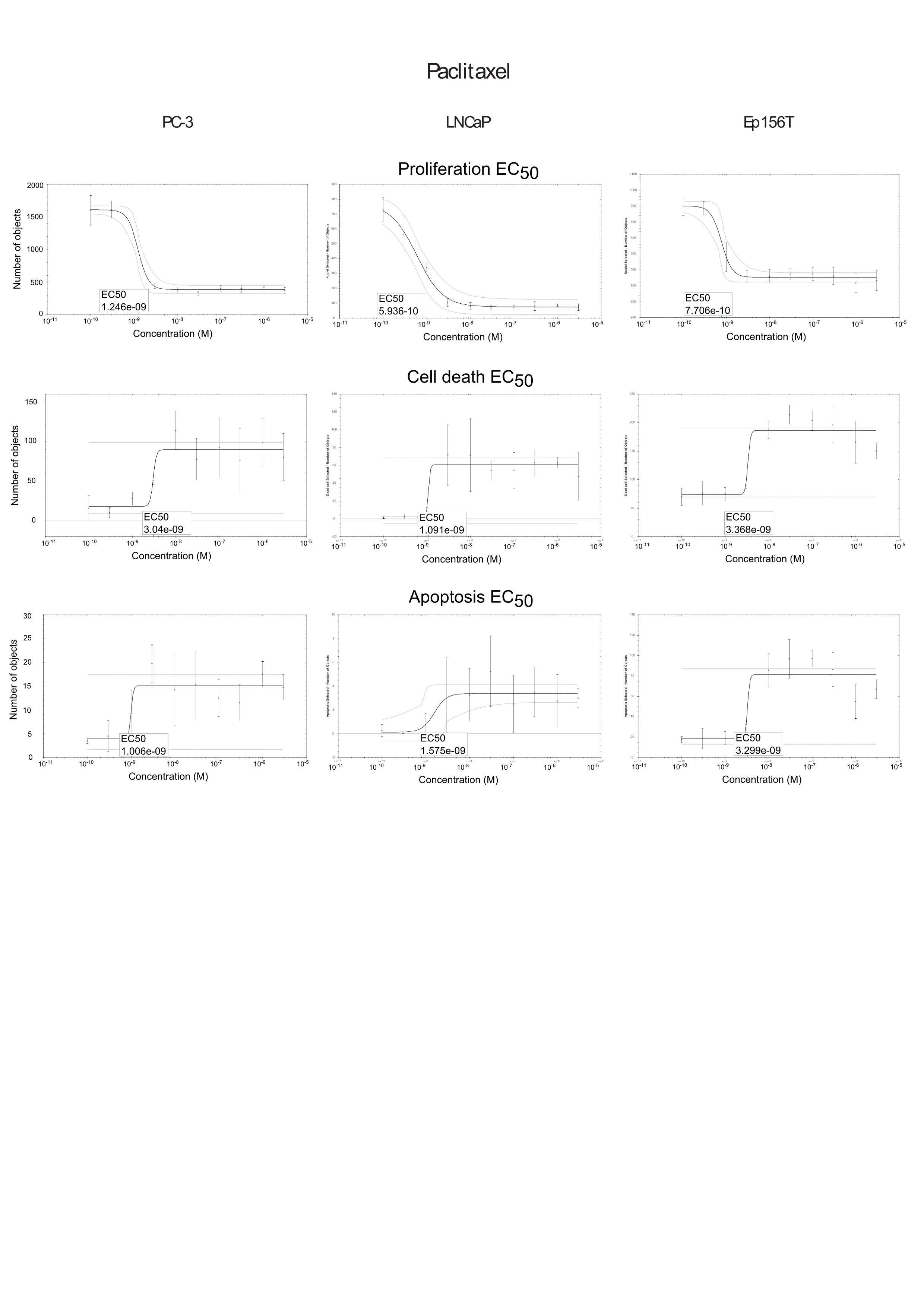

Supplement: S7 Fig — EC50values, calculated with PerkinElmer Harmony software, are displayed in each graph. (TIF) [file pone.0126111.s007.tif]

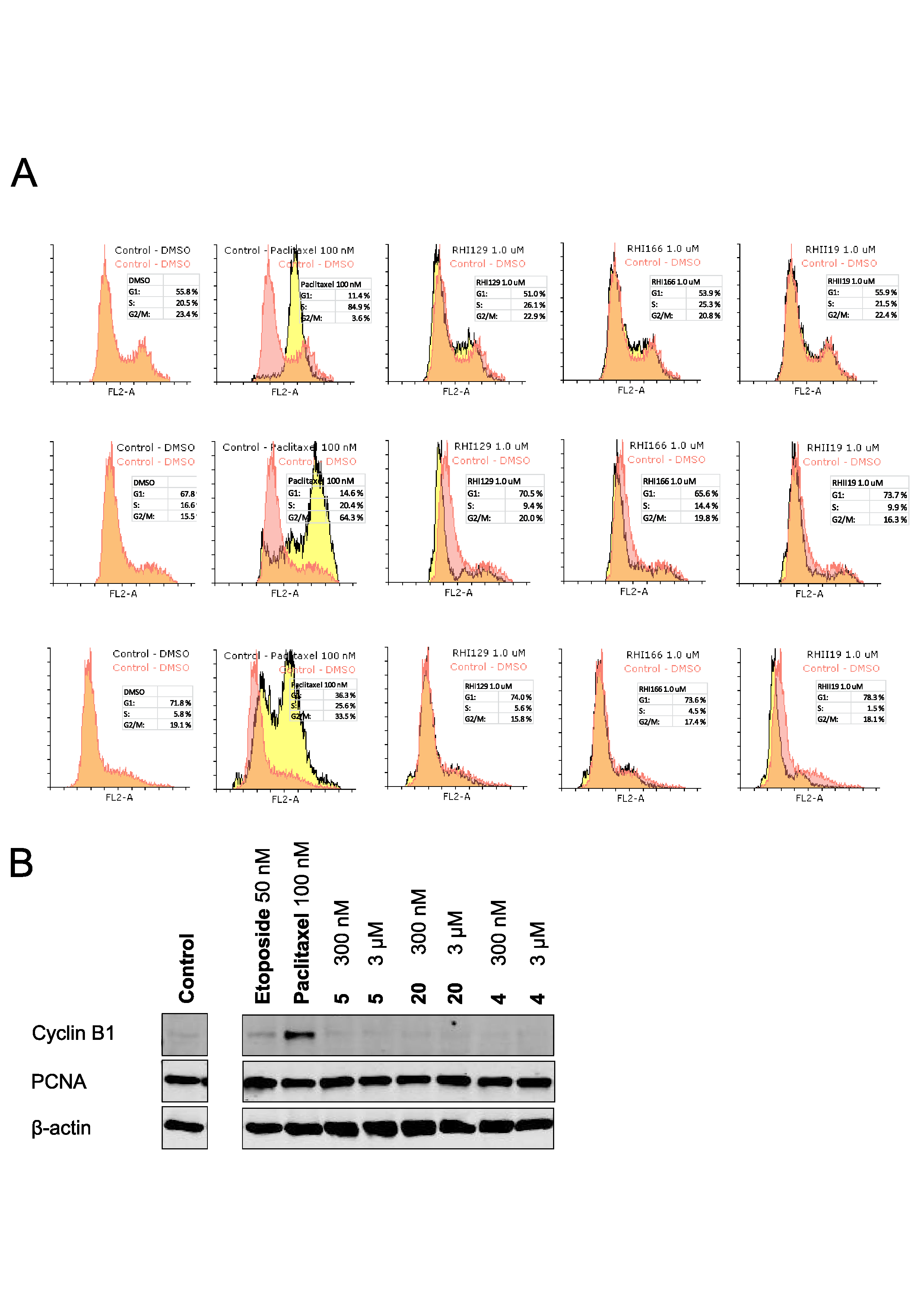

Supplement: S8 Fig — A) Histograms for DNA content for the cell lines PC-3, LNCaP and Ep156T; exposed to three betulin derivatives 4, 5 and 20 at 1 μM concentration for 72 h in monolayer culture. Relative proportions of each cell cycle phase (G1, S and G2), assessed using Flowing software (v2.5.1), are displayed next to each histogram (in %). B) Expression of proliferating cell nuclear antigen (PCNA) and mitotic cyclin B1 protein in response to 72h exposure to betulin derivatives. 24h paclitaxel treatment was used as mitotic arrest control. (TIF) [file pone.0126111.s008.tif]

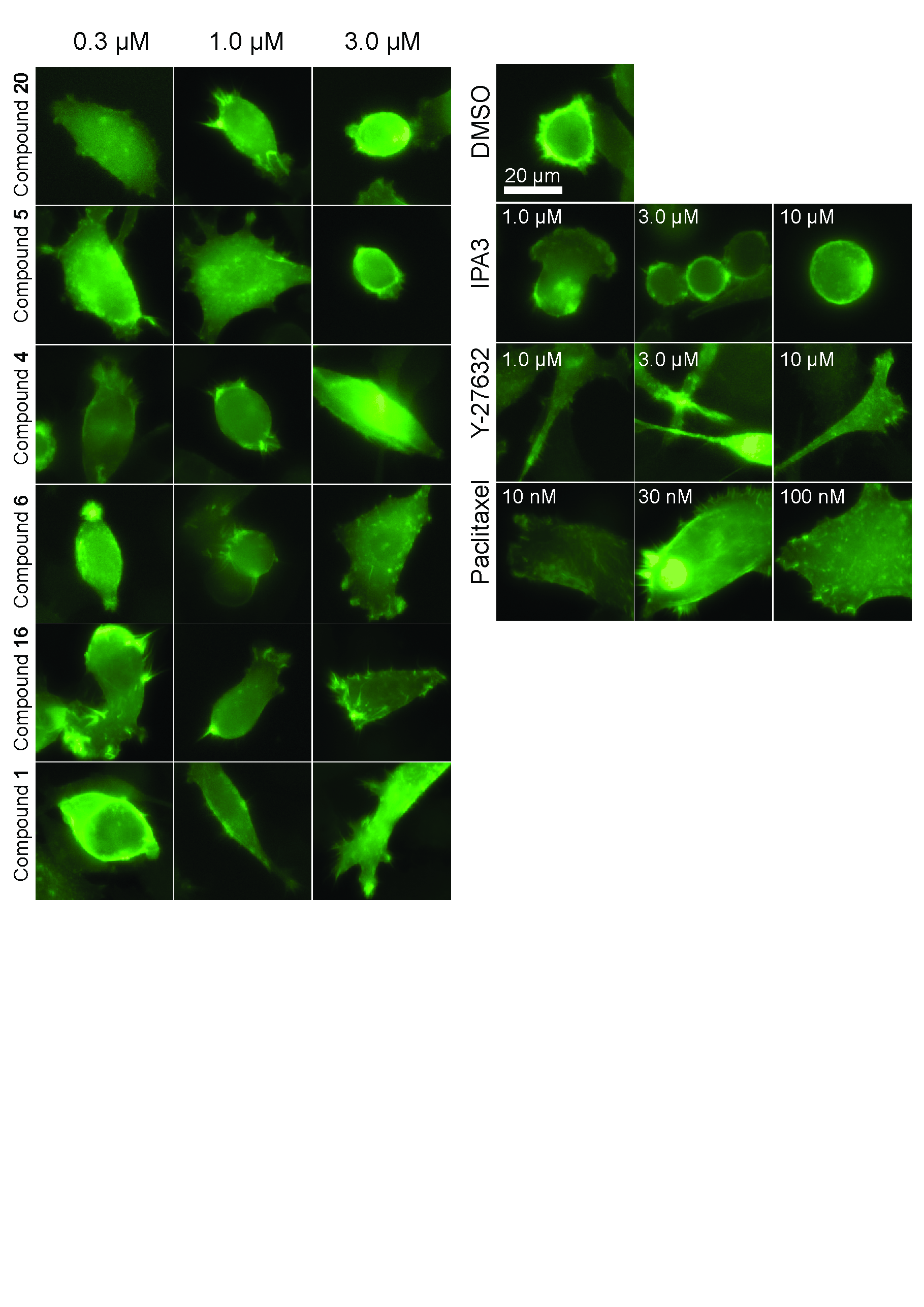

Supplement: S9 Fig — Actin cytoskeleton (filamentous or F- actin) is stained green (LifeAct), nuclei with a red dye (confocal microscope images, 40× objective, scale bar shown for each panel on the right lower corner). (TIF) [file pone.0126111.s009.tif]
