# Supplementary material for: Optimization of Invasion-Specific Effects of Betulin Derivatives on Prostate Cancer Cells through Lead Development
Source: PLoS One. 2015 May 12;10(5):e0126111. doi: 10.1371/journal.pone.0126111 (PMC4428838; doi:10.1371/journal.pone.0126111)
Supplement: S1 File — (DOCX) [file pone.0126111.s010.docx]

Supporting Information for

**Optimization of Invasion-specific Effects of Betulin Derivatives on Prostate Cancer Cells through Lead Development**

Ville Härmä^1^¶, Raisa Haavikko^2^¶, Johannes Virtanen^1^, Ilmari Ahonen^3^, Hannu-Pekka Schukov^3^, Sami Alakurtti^4^, Enkhee Purev^1,6^, Heiko Rischer^5^, Jari Yli-Kauhaluoma^2^, Vânia M. Moreira^2,†^ Matthias Nees ^1,3&^, Kirsi-Marja Oksman-Caldentey^5&^

^1^Industrial Biotechnology, VTT Technical Research Centre of Finland, Turku, Finland; ^2^Division of Pharmaceutical Chemistry and Technology, Faculty of Pharmacy, University of Helsinki, Helsinki, Finland; ^3^Turku Centre for Biotechnology BTK, University of Turku, Turku, Finland; ^4^Process Chemistry and Environmental Engineering, VTT Technical Research Centre of Finland, Espoo, Finland; ^5^Industrial Biotechnology, VTT Technical Research Centre of Finland, Espoo, Finland; ^6^ National University of Mongolia, Ulanbataar, Mongolia

*Corresponding author

E-mail: [kirsi-marja.oksman@vtt.fi](mailto:kirsi-marja.oksman@vtt.fi) (K-MO)

^¶^These authors contributed equally to this work.

^&^These authors also contributed equally to this work.

**SUPPLEMENTARY MATERIALS AND METHODS**

**Chemistry**

*General experimental details*

Commercially available reagents were used without further purification: all solvents were anhydrous and HPLC grade, purchased from Sigma-Aldrich (St. Louis, MO, USA). All reactions were performed in oven-dried glassware under an inert atmosphere of dry argon. Thin layer chromatography (TLC) was performed on E. Merck Silica Gel 60 aluminium packed plates, visualization accomplished by UV illumination and staining with 5% H_2_SO_4_ in MeOH. ^1^H NMR spectra were recorded on a Varian Mercury-VX 300 MHz with chemical shifts reported as parts per million (in CDCl_3_ at 23 °C, solvent peak at 7.26 ppm as an internal standard). ^13^C NMR spectra were obtained on a Varian Mercury-VX 75 MHz spectrometer with chemical shifts reported as parts per million (CDCl_3_ at 23 °C, solvent peak at 77.0 ppm as an internal standard). Elemental analyses (CHN) were obtained from Robertson Microlit Laboratories, Inc., Madison, New Jersey, USA.

*Compounds*

Crude betulin was recrystallized from 2-propanol/H_2_O azeotrope to yield 99% pure betulin as a white solid. Betulonic acid was obtained from betulin by Jones oxidation, and used as a versatile intermediate for chemical syntheses. A total of 75 betulin derivatives were synthesized from using betulin and betulinic and betulonic acids as starting materials (Figures 1 – 6 in this section). The synthesis of compounds **4**, **5**, **6**, **7**, **8**, **9**, **10**, **15**, **16**, **19**, **20**, **22**, **23**, and **24** is described in our previous study.[1] The synthesis of compounds **12**, **11**, **17**, and **21** is described in our previous work.[2] The synthesis of **13** and **14** has been previously described.[3] Compound **25** has also been previously reported.[4] The synthesis of **1**, **2**, **and 3** has been reported.[5] The synthesis of other betulin derivatives is described in detail in our previous publications.[6-8] The synthesis of **18** and **MPi-I-077** followed the procedure described below. The abietanes derivatives used in this work are depicted on Figure 7. Synthesis of abietane urea derivatives **28** – **32** has been reported elsewhere.[9] Synthesis of the rest abietane derivatives will be described in details elsewhere.

**Lupa-2,20(29)-dieno[2,3-*b*]pyrazino-28-nitrile** (**18**)

A mixture of betulonic acid (0.20 g, 0.44 mmol), 1,2-diaminoethane (130 mg, 2.0 mmol), sulfur (130 mg, 4.1 mmol) and morpholine (4 mL) was refluxed for 21 h. Water was added, and the resulting mixture was extracted with CH_2_Cl_2_. The organic phase was washed with 1 M hydrochloric acid, water, a saturated aqueous solution of NaHCO_3_, water and brine, dried over anhydrous Na_2_SO4 and evaporated. The crude product was purified with SiO_2_ column chromatography (20-50% EtOAc/*n*-hexane) to give lupa-2,20(29)-dieno[2,3-*b*]pyrazin-28-oic acid as a white crystalline solid (147 mg, 68%). ^1^H NMR (300 MHz, CDCl_3_) *δ* 8.42 (1H, d, *J* = 2.4 Hz), 8.29 (1H, d, *J* = 2.4 Hz), 4.76 (s, 1H), 4.64 (s, 1H), 3.05 (m, 2H), 2.46 (1H, d, *J* = 16.5 Hz), 2.29 (m, 2H), 1.30 (s, 3H), 1.72 (s, 3H), 1.27(s, 3H), 1.04 (s, 3H), 1.03 (s, 3H), 0.81 (s, 3H); ^13^C NMR (75 MHz, CDCl_3_) *δ* 181.5, 160.0, 151.0, 150.5, 142.6, 141.5, 110.0, 56.6, 53.2, 49.4, 49.0, 48.7, 47.1, 42.7, 40.8, 39.7, 38.7, 37.3, 37.0, 33.6, 32.4, 31.7, 30.8, 30.0, 25.7, 24.2, 21.6, 20.3, 19.7, 16.4, 15.9, 14.9.

A mixture of lupa-2,20(29)-dieno[2,3-*d*]pyrazin-28-oic acid (140 mg, 0.28 mmol), oxalyl chloride (44 mg, 0.34 mmol), and a drop of DMF in dry THF (10 mL) was stirred at room temperature for 2 h. The solvent was evaporated, and the residue was dissolved in EtOAc. The organic phase was washed with a saturated aqueous solution of NaHCO_3_, water and brine, dried over anhydrous Na_2_SO4 and evaporated. The resulting crude lupa-2,20(29)-dieno[2,3-*b*]pyrazin-28-oyl chloride was dissolved in CHCl_3_ (5 mL), and an aqueous solution of 25% ammonia (2 mL) was added to the mixture. The resulting solution was stirred at room temperature for 30 min and evaporated to dryness to give lupa-2,20(29)-dieno[2,3-*d*]pyrazin-28-oic amide as a white crystalline solid (137 mg, quant.). ^1^H NMR (300 MHz, CDCl_3_) *δ* 8.46 (s, 1H), 8.29 (d, *J* = 2.4 Hz, 1H), 5.30 (br s, 2H), 4.77 (s, 1H), 4.64 (s, 1H), 3.72 (q, *J* = 7.0 Hz, 1H), 3.12 (d, *J* = 17.0 Hz, 2H), 2.67–2.40 (m, 2H), 2.08–1.74 (m, 5H), 1.71 (s, 3H), 1.67–1.35 (m, 13H), 1.31 (s, 3H), 1.30 (s, 3H), 1.04 (s, 3H), 1.03 (s, 3H), 0.82 (s, 3H); ^13^C NMR (75 MHz, CD_3_OD) *δ* 182.4, 161.4, 152.3, 152.0, 143.8, 142.3, 110.0, 57.1, 54.3, 51.2, 50.1, 48.1, 43.7, 41.9, 40.62, 39.4, 39.1, 37.9, 34.6, 34.3, 31.9, 31.8, 30.7, 27.0, 24.4, 22.8, 21.2, 19.6, 16.6, 16.3, 15.0; IR (ν, cm^-1^): 886, 1107, 1184, 1402, 1665, 2869, 2948, 3044, 3129; HRMS: *m/z* calcd for C_32_H_48_N_3_O 489.3797, found 490.3796 [M+H]^+^.

Lupa-2,20(29)-dieno[2,3-*d*]pyrazin-28-oic amide (88 mg, 0.18 mmol) in DMF (5 mL) was cooled to water-ice bath temperature and cyanuric chloride (30 mg, 0.16 mmol) was added to the solution. After stirring for 1 h, water was added, and the resulting mixture was extracted with EtOAc. The organic phase was washed with water and brine, dried over anhydrous Na_2_SO_4_ and evaporated. The crude product was purified with SiO_2_ column chromatography (5-20% EtOAc/*n*-hexane) to give **18** as a white crystalline solid (35 mg, 41%). ^1^H NMR (300 MHz, CDCl_3_) *δ* 8.41 (d, *J* = 2.4 Hz, 1H), 8.27 (d, *J* = 2.4 Hz, 1H), 4.80 (s, 1H), 4.70 (s, 1H), 3.04 (d, *J* = 16.5 Hz, 1H), 2.78–2.61 (m, 1H), 2.46 (d, *J* = 16.5 Hz, 1H), 2.30–2.14 (m, 1H), 2.16–1.91 (m, 4H), 1.84 (m, 1H), 1.71 (s, 3H), 1.68–1.56 (m, 5H), 1.52 (m, 6H), 1.45–1.35 (m, 3H), 1.31 (s, 3H), 1.30 (s, 3H), 1.17 (s, 3H), 1.01 (s, 3H), 0.83 (s, 3H). ^13^C NMR (75 MHz, CDCl_3_) *δ* 159.7, 150.6, 148.0, 142.4, 141.5, 123.4, 111.0, 53.1, 49.1, 48.8, 48.6, 42.4, 41.4, 40.5, 39.5, 36.8, 35.8, 33.5, 31.5, 31.1, 29.6, 29.1, 25.1, 24.0, 21.3, 20.1, 19.5, 16.2, 15.6, 14.9. HRMS: *m/z* calcd for C_32_H_46_N_3_: 472.3692, found 472.3701 [M+H]^+^. Elemental analysis. calcd for C_32_H_45_N_3_ C 81.48; H 9.62; N 8.91, found C 78.08 %, H 9.66%, N 8.82%.

**Lup-20(29)-ene-3,28-diol, 3,28-bis[3-(4-nitrophenyl)-(*E*)-propen-2-oate] (26).**

Betulin (15.0 g, 33.9 mmol), *p*-nitrocinnamoyl chloride (15.8 g, 74.7 mmol) and *N,N*-dimethylaminopyridine (9.11 g, 74.6 mmol, DMAP) were dissolved in pyridine (300 mL). Reaction mixture was stirred at 40 °C. Reaction was monitored with TLC (1:3 EtOAc/*n*-hexane). After 2 d the formed precipitate was filtered and pyridine was evaporated. Residual solid was dissolved in toluene (900 mL) and organic layer was washed with 5% HCl (3 × 500 mL), water (400 mL), saturated NaHCO_3_ (2 × 500 mL) and water (400 mL). Organic layer was dried with anhydrous Na_2_SO_4_ and solvents were evaporated. Residue was dried in an oven to yield a mixture of monoester and diester (17.2 g, 21.7 mmol, 64%). A mixture of monoester and diester (15.2 g), *p*-nitrocinnamoyl chloride (11.4 g, 53.9 mmol) and DMAP (6.0 g, 49.1 mmol) in pyridine (200 mL) was stirred at 40 °C. Reaction was monitored with TLC (1:3 EtOAc/*n*-hexane). After 1 d pyridine was evaporated and the residual precipitate was dissolved in toluene (400 mL), washed with 5% HCl (3 × 300 mL), water (400 mL), saturated NaHCO_3_ (2 × 400 mL) and water (400 mL). Organic phase was dried with anhydrous Na_2_SO_4_. Solvent was evaporated and the precipitate was washed with acetone and dried in oven. to give a yellow solid (9.00 g, 11.4 mmol, 34%). ^1^H NMR (500 MHz, CDCl_3_) *δ* 8.28-8.22 (4H, m), 7.75-7.65 (6H, m), 6.58 (2H, dd, *J* = 16.03, 13.83 Hz), 4.67 (2H, d, *J* = 4.67 Hz), 4.67-4.60 (1H, m), 4.46 (1H, d, *J* = 10.40 Hz), 4.01 (1H, d, *J* = 11.05 Hz), 2.57-2.46 (3H, m), 2.08-1.95 (1H, m), 1.95-1.81 (2H, m), 1.71 (6H, s), 1.69-1.58 (3H, m), 1.57-1.52 (1H, m), 1.49-1.38 (5H, m), 1.37- 1.31 (2H, m), 1.19-1.13 (1H, m), 1.08 (3H, s), 1.05-1.00 (6H, m), 0.93 (3H, s), 0.90 (3H, s), 0.89 (3H, s). ^13^C NMR (125 MHz, CDCl_3_) *δ* 166.5, 165.9, 150.0, 148.5, 148.4, 141.7, 141.3, 140.7, 140.5, 128.7, 128.6, 124.2, 123.1, 122.5, 110.0, 81.7, 63.4, 55.4, 50.3, 48.8, 47.7, 46.6, 42.7, 40.9, 38.4, 38.0, 37.6, 37.1, 34.6, 34.1, 29.8, 29.5, 28.0, 27.1, 25.1, 23.7, 20.8, 19.1, 18.6, 16.7, 16.2, 16.0, 14.8. HRMS: *m/z* calcd for C_48_H_60_N_2_NaO_8_ 815.4247, found 815.4242 [M+Na]^+^.

#### SUPPLEMENTARY FIGURES

**Figure 1**. Betulinic acid (**1**) and betulonic acid (**2**) derivatives.

**Figure 2**. Betulin derivatives (cont.).

**Figure 3**. Betulin derivatives (cont.).

**Figure 4**. Betulin derivatives (cont.).

**Figure 5**. Betulin derivatives (cont.).

**Figure 6**. Betulin derivatives (cont.).

**Figure 7**. Abietane derivatives.

**Figure 8**. ^1^H-NMR of compound **18** recorded in CDCl_3_.

**Figure 9**. ^13^C-NMR of compound **18** recorded in CDCl_3_.

**
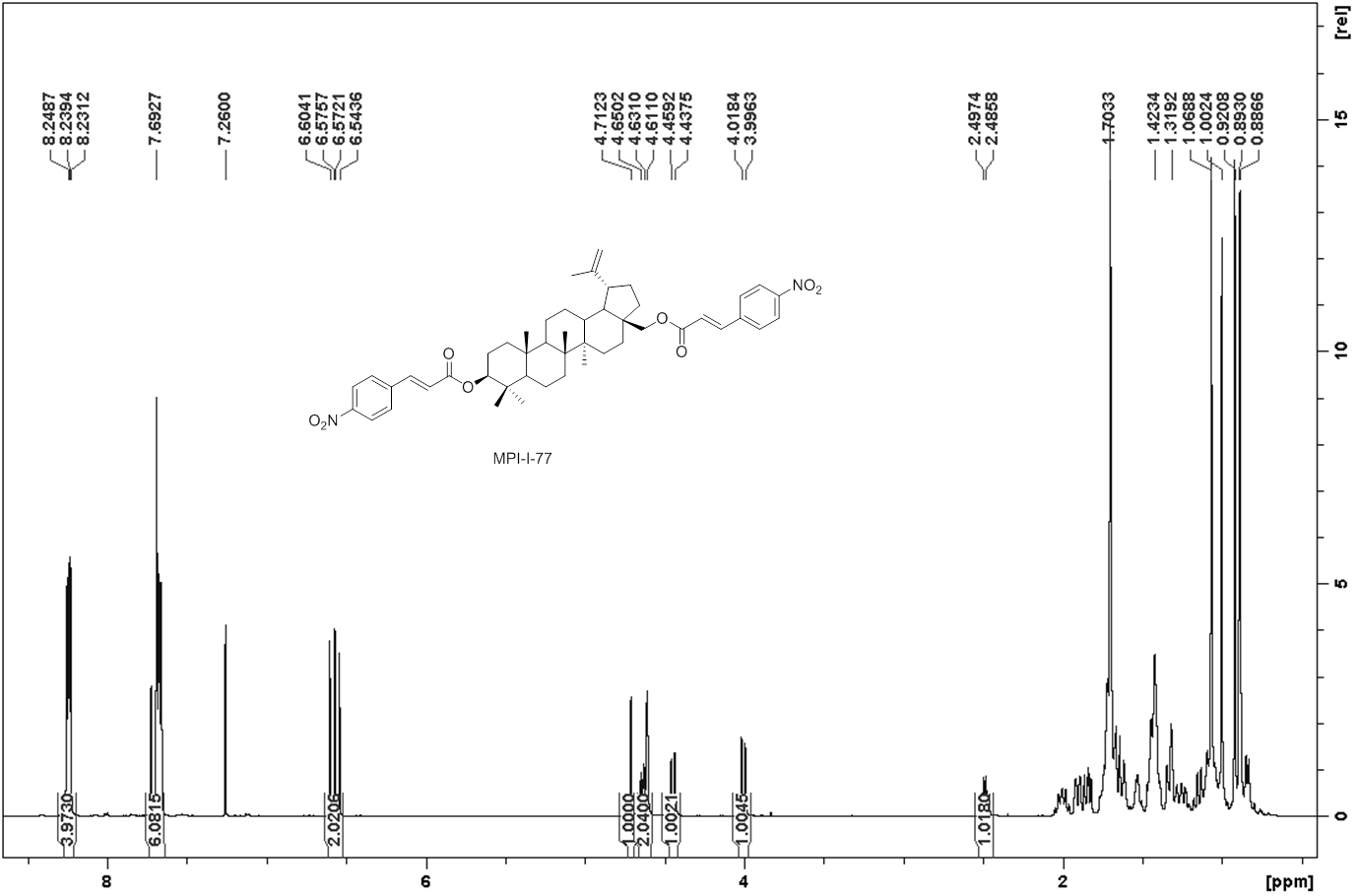
Figure 10**. ^1^H-NMR of compound **26** recorded in CDCl_3_.

**
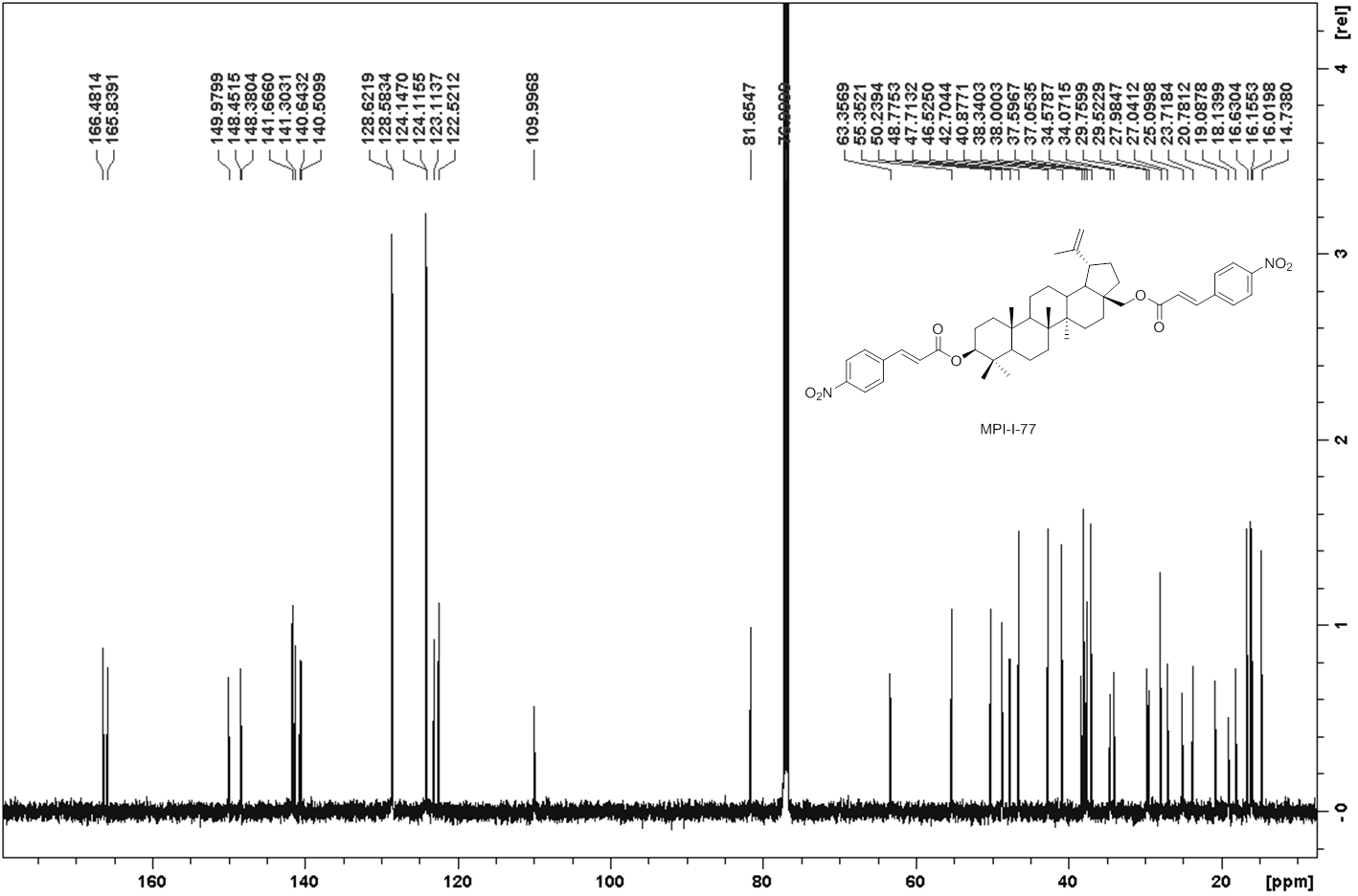
Figure 11**. ^13^C-NMR of compound **26** recorded in CDCl_3_.

**Automated Image Analysis & Statistics**

**Morphometric image analysis, data normalization, and mathematical/statistical modelling.** Automated image analyses were essentially performed as previously described.[10] Specific statistical methods utilized for data processing and mathematical modelling of treatment responses are described below.

#### Morphometric and normalization of image data sets. Structures smaller than 200 pixels or partially outside the image boundaries were removed. Images of poor quality were removed. Unless otherwise specified, only the green (Calcein AM) channel was used. To measure the effects of drug treatments on 3D spheroids, we modelled the median log of structure size (=Area), invasive structures (=Complexity) and dead cells (= the proportion of red color in the images) as quantitative responses. To be able to compare all treatments and readout unanimously, models were fitted on the entire dataset with 1440 rows, each corresponding to a single image. The median log(Area) and median log(Complexity) were modelled using linear mixed models that in addition to the treatment effects took into account other possible sources of variation:

1) Each treatment-concentration combination was treated as a separate level and categorical variable, and thus modelled with a unique fixed effect. An alternative analysis would model the concentration as a continuous variable, assuming a predefined shape for the dose-response such as linear or quadratic. However, we have no reason to assume such a consistent relationship between doses and effects and also do not have the number of dose-levels to truly benefit from such assumptions. Thus, we allowed the effects for treatment-dose combinations to be estimated freely.

2) Replicates: Treatments were replicated in both multiple wells and multiple plates which allowed us to estimate both the well-to-well and plate-to-plate variation using the data. This was achieved using random effects for plate and well effects.

3) Normalization: Due to practical reasons, many of the procedures in the experiment set were performed in systematic order, row by row, across the wells in the plates, starting from one corner and ending in the opposite one. Such procedures occur in all phases of the experiment, from the initial cell culturing to feeding and finally, imaging. Thus, there is a possibility that the well position in the plate can induce a trend to our responses. To account for this, we included well-number as a continuous covariate to the model and defined its interaction term with the plate effect to allow the possible slopes to differ across plates.

4) Both the random effects and the residual term were assumed to be normally distributed, with a mean of zero. These models were then fitted separately for all cell lines and compound responses, using REML (restricted maximum likelihood) estimation.

The effect of well’s position in the plate was found to be negligible across all models and also statistically non-significant in six out of eight models. We could conclude that the possible trend induced by the systematic aspects in the experiment did not have a significantly large effect on the study results. This statement was further supported by the fact that all models demonstrated convergence problems in the fitting phase, which is often related to certain model parameters being too small to be uniquely estimable. The well-number effect was then dropped from all models which also erased all convergence issues. The estimated treatment effects were extracted and their confidence intervals calculated, based on normal approximation.

**Scaling of compound responses.** All compound effects were scaled based on the control treatment DMSO and the most effective paclitaxel dose. This was done by defining the effect of DMSO control as zero, and that of paclitaxel’s as the 100% values (maximum change). This way, we achieved effect size estimates that were comparable between all cell lines, and compound responses, and interpretable in relation to DMSO and paclitaxel. These estimates were also scaled based on DMSO and paclitaxel, using the same procedure as above (data not shown). Most of the time the estimated residual variation, which in this case is the within-well variation in response, was larger than both of the well-to-well and plate-to-plate variation. All estimates were fairly small compared to the largest treatment effects but not insignificant. We can conclude, that there is evidence of both random well-to-well and plate-to-plate variation, which was however considerably smaller in magnitude than the effects of the most potent treatments.

#### References

References

1. Haavikko R, Nasereddin A, Sacerdoti-Sierra N, Kopelyanskiy D, Alakurtti S, et al. (2014) Heterocycle-fused lupane triterpenoids inhibit *Leishmania donovani* amastigotes. Med Chem Commun 5: 445-451.

2. Parkkari T, Haavikko R, Laitinen T, Navia-Paldanius D, Rytilahti R, et al. (2014) Discovery of triterpenoids as reversible inhibitors of alpha/beta-hydrolase domain containing 12 (ABHD12). PLoS One 9: e98286. 10.1371/journal.pone.0098286 [doi].

3. Antimonova AN, Uzenkova NV, Petrenko NI, Shakirov MM, Shul’ts EE, et al. (2008) Synthesis of betulonic acid amides. Chem Nat Compd 44: 327-333.

4. Safe SH, Chintharlapalli S. (2008) .

5. Pohjala L, Alakurtti S, Ahola T, Yli-Kauhaluoma J, Tammela P. (2009) Betulin-derived compounds as inhibitors of alphavirus replication. J Nat Prod 72: 1917-1926. 10.1021/np9003245 [doi].

6. Alakurtti S, Heiska T, Kiriazis A, Sacerdoti-Sierra N, Jaffe CL, et al. (2010) Synthesis and anti-leishmanial activity of heterocyclic betulin derivatives. Bioorg Med Chem 18: 1573-1582. [http://dx.doi.org/10.1016/j.bmc.2010.01.003](http://dx.doi.org/10.1016/j.bmc.2010.01.003).

7. Alcazar W, López AS, Alakurtti S, Tuononen M, Yli-Kauhaluoma J, et al. (2014) Betulin derivatives impair leishmania braziliensis viability and host–parasite interaction. Bioorg Med Chem 22: 6220-6226. [http://dx.doi.org/10.1016/j.bmc.2014.08.023](http://dx.doi.org/10.1016/j.bmc.2014.08.023).

8. Salin O, Alakurtti S, Pohjala L, Siiskonen A, Maass V, et al. (2010) Inhibitory effect of the natural product betulin and its derivatives against the intracellular bacterium chlamydia pneumoniae. Biochem Pharmacol 80: 1141-1151. [http://dx.doi.org/10.1016/j.bcp.2010.06.051](http://dx.doi.org/10.1016/j.bcp.2010.06.051).

9. Fallarero A, Skogman M, Kujala J, Rajaratnam M, Moreira VM, et al. (2013) (+)-Dehydroabietic acid, an abietane-type diterpene, inhibits staphylococcus aureus biofilms in vitro. Int J Mol Sci 14: 12054-12072.

10. Harma V, Schukov HP, Happonen A, Ahonen I, Virtanen J, et al. (2014) Quantification of dynamic morphological drug responses in 3D organotypic cell cultures by automated image analysis. PLoS One 9: e96426. 10.1371/journal.pone.0096426 [doi].
